# Structure of a fully assembled tumor-specific T cell receptor ligated by pMHC

**DOI:** 10.1016/j.cell.2022.07.010

**Published:** 2022-08-18

**Authors:** Lukas Sušac, Mai T. Vuong, Christoph Thomas, Sören von Bülow, Caitlin O’Brien-Ball, Ana Mafalda Santos, Ricardo A. Fernandes, Gerhard Hummer, Robert Tampé, Simon J. Davis

**Affiliations:** 1 Institute of Biochemistry, Biocenter, Goethe University Frankfurt, Max-von-Laue-Str. 9, 60438 Frankfurt am Main, Germany; 2 Radcliffe Department of Medicine, John Radcliffe Hospital, University of Oxford, Oxford OX3 9DS, UK; 3 Medical Research Council Human Immunology Unit, John Radcliffe Hospital, University of Oxford, Oxford OX3 9DS, UK; 4 Department of Theoretical Biophysics, Max Planck Institute of Biophysics, Max-von-Laue-Str. 3, 60438 Frankfurt am Main, Germany; 5 Institute of Biophysics, Goethe University Frankfurt, Max-von-Laue-Str. 1, 60438 Frankfurt am Main, Germany

**Keywords:** adaptive immunity, antigen presentation, electron microscopy, membrane proteins, receptor triggering, structural cell biology, supramolecular complexes

## Abstract

The T cell receptor (TCR) expressed by T lymphocytes initiates protective immune responses to pathogens and tumors. To explore the structural basis of how TCR signaling is initiated when the receptor binds to peptide-loaded major histocompatibility complex (pMHC) molecules, we used cryogenic electron microscopy to determine the structure of a tumor-reactive TCRαβ/CD3δγε_2_ζ_2_ complex bound to a melanoma-specific human class I pMHC at 3.08 Å resolution. The antigen-bound complex comprises 11 subunits stabilized by multivalent interactions across three structural layers, with clustered membrane-proximal cystines stabilizing the CD3-εδ and CD3-εγ heterodimers. Extra density sandwiched between transmembrane helices reveals the involvement of sterol lipids in TCR assembly. The geometry of the pMHC/TCR complex suggests that efficient TCR scanning of pMHC requires accurate pre-positioning of T cell and antigen-presenting cell membranes. Comparisons of the ligand-bound and unliganded receptors, along with molecular dynamics simulations, indicate that TCRs can be triggered in the absence of spontaneous structural rearrangements.

## Highlights

• Connecting peptides create rigid links to stabilize membrane-bound TCR subunits
• Sterol lipid contributes to transmembrane assembly of the TCR complex
• A pMHC/TCR structure highlights the cooperative nature of antigen recognition
• TCR signaling may be triggered without spontaneous structural rearrangements

## Graphical abstract

**Figure:**
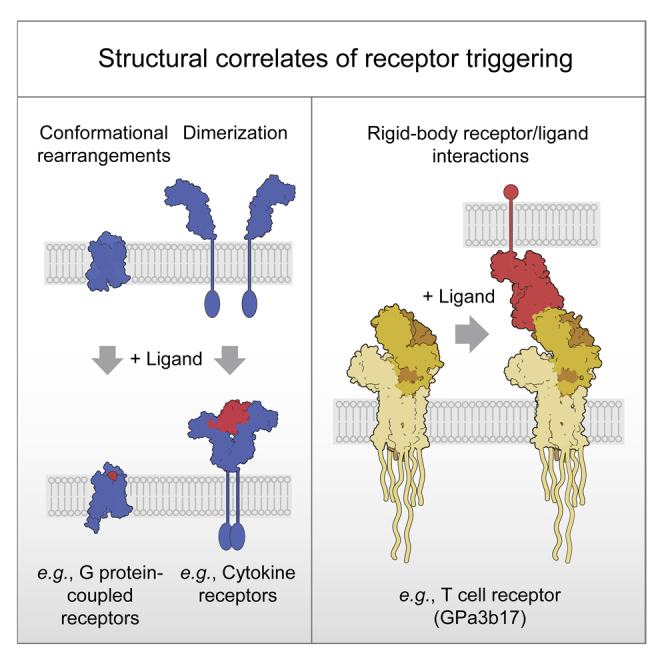

## Teaser

Cryo-EM structural analysis of a T cell receptor (TCR) complex bound to a tumor-specific human class I pMHC indicates the functional impact of connecting peptides and sterol lipid for complex assembly and suggests that TCR signaling may be triggered without spontaneous structural rearrangements.

## Introduction

To provide host protection, the immune system must deal with all comers. For T cells, which orchestrate the adaptive response, the solution is to render down pathogens and tumors to short (up to 24-residue) peptides for presentation to T cell receptors (TCRs) as complexes with major histocompatibility complex (MHC) proteins (peptide-loaded MHC [pMHC]). Despite decades of research, how the TCR initiates T cell signaling after binding peptide-loaded MHC class I or II molecules remains one of the great enigmas of immunology (Courtney et al., 2018; Mariuzza et al., 2020). The TCR belongs to the immunoglobulin superfamily of cell surface proteins and comprises complexes of clonotypic antigen-binding αβ heterodimers, each consisting of variable (V) and constant (C) domains, associated in a 1:1:1:1 ratio with invariant CD3-εδ, CD3-εγ, and CD3-ζζ dimers essential for signal transduction (Call et al., 2004; Punt et al., 1994). Although many receptors for soluble ligands, e.g., growth factor receptors, are triggered by autophosphorylation, the TCR lacks associated kinase activity. Instead, after pMHC binding, the TCR is phosphorylated on tyrosine residues in its intrinsically unstructured cytosolic region by extrinsic Src-family kinases (Straus and Weiss, 1992). Despite being among the most-studied membrane receptors (Mariuzza et al., 2020), it is not yet understood how ligand-binding results in TCR triggering.

TCRs diffuse on the cell surface as individual proteins poised to initiate signaling upon encountering single pMHC complexes (Brameshuber et al., 2018; James et al., 2007). Given this constraint, it has been widely anticipated that TCR signaling would be initiated by spontaneous allosteric rearrangements. Using environment-sensitive fluorescent probes and structural analyses, a candidate conformational change in the AB loop of Cα triggered by pMHC engagement has been observed (Beddoe et al., 2009). Nuclear magnetic resonance (NMR) studies and molecular dynamics (MD) simulations identified putative small-amplitude structural rearrangements also involving the Cα AB loop, along with two additional sites: the Cβ FG loop projecting from the Vβ/Cβ interface and several Cβ residues at or close to the Cβ αA helix forming part of the CD3 docking site (Rangarajan et al., 2018). Other NMR analyses suggested that conformational remodeling of the Cβ H3 helix region at the membrane-proximal face of the TCR accompanies pMHC binding (Natarajan et al., 2017). Crystal structural studies of soluble unbound and liganded TCR-αβ ectodomains, on the other hand, offer little support for allosteric effects since, typically, only minor adjustments in the ligand-binding site have been observed (Rudolph et al., 2006).

Other explanations for receptor signaling allow rigid-body interactions of TCR-αβ with pMHC and for structural changes to occur elsewhere, either spontaneously or under force. It is suggested, for example, that CD3-ζζ loses cohesion with TCR-αβ (Lanz et al., 2021) or that the membrane-proximal cytosolic regions of the CD3-ζ subunits form a closer association (Lee et al., 2015), allowing signaling. Evidence that the TCR might function as a mechanosensor has come from experiments in which tangential forces applied to the receptor were observed to initiate calcium signaling (Kim et al., 2009). It was suggested that anisotropic forces created by the directional scanning of antigen-presenting cells (APCs) by T cells displaces TCR-αβ relative to the CD3 subunits, leading to signaling (Lee et al., 2015). Finally, a mechanism has been proposed wherein signaling does not rely on ligand-induced structural rearrangements in the assembled complex, of any type. According to this concept, referred to as the “kinetic-segregation” model, pMHC binding prevents the diffusion of the receptor out of regions of close contact between T cells and APCs that favor signaling. These regions are proposed to favor signaling because they would be depleted of phosphatases such as CD45 that are larger than pMHC/TCR complexes and counteract the activities of kinases (Davis and van der Merwe, 1996, 2006). An understanding of the structural similarities or differences between fully assembled unliganded and pMHC-bound TCRs would dramatically reduce the number of possible explanations for how signaling is initiated by the TCR.

The recent cryogenic electron microscopy (cryo-EM)-based structure of a non-clonotypic, unliganded TCR disclosed the overall arrangement of the TCR-αβ/CD3 subunits (Dong et al., 2019). Here, we took advantage of a high-affinity, tumor-reactive TCR to obtain the structure of a fully assembled ligand-bound receptor. Our 3.08 Å cryo-EM structure establishes the structural basis of CD3-εδ and CD3-εγ heterodimer stabilization and an unexpected role for lipids in TCR assembly, and reveals that antigen recognition by the receptor relies on the cooperative effects of small adhesive proteins. Most importantly, despite some minor conformational rearrangements, the structure reveals that the TCR is largely unchanged by ligand binding unlike most, if not all other classes of signaling receptors.

## Results

### Trapping and isolation of the TCR from the cell surface using a cognate pMHC

**Figure S1:**
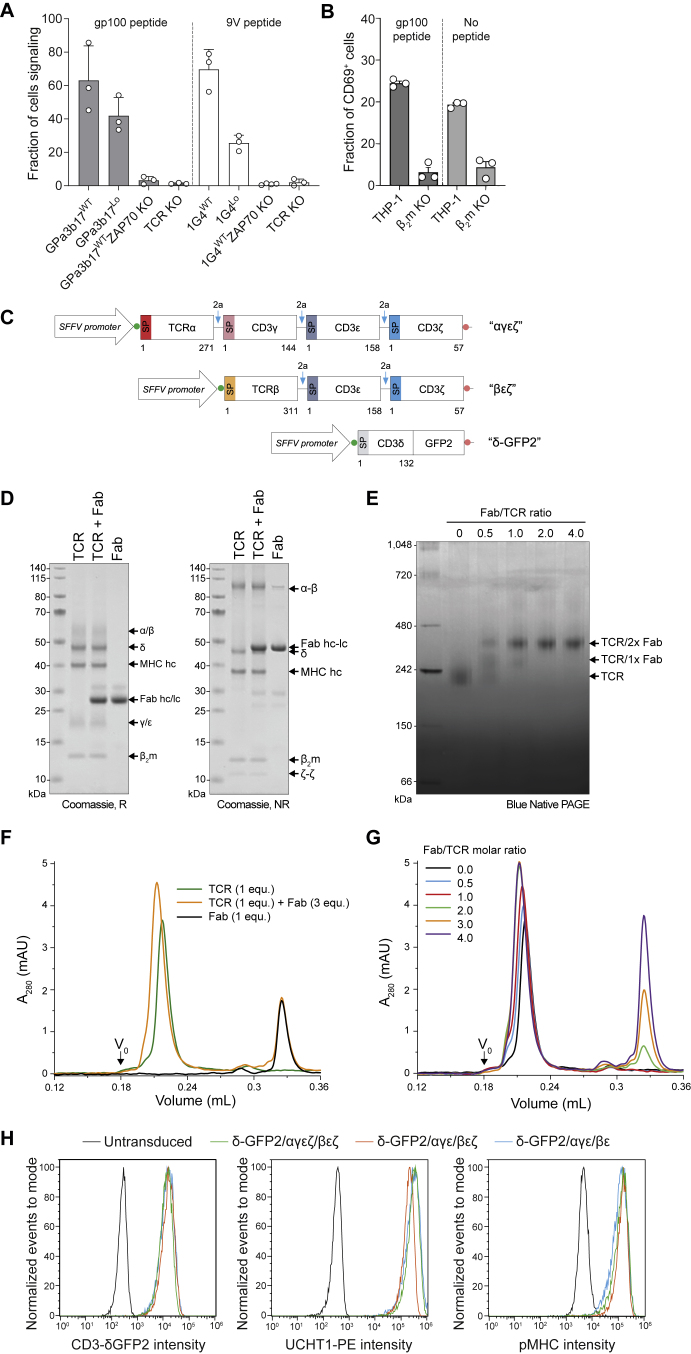
Signaling capacity, expression, and isolation of stoichiometrically defined TCR-CD3/pMHC complexes, related to STAR Methods (A) Calcium signaling on SLBs. Jurkat T cells, with endogenous TCR genes inactivated using CRISPR-Cas9, were transfected with cDNA encoding the αβ subunits of the tumor-reactive, affinity-matured GPa3b17 TCR (*K*_D_ = 13 pM; Liddy et al., 2012). The 1G4 TCR binds to the 9V variant of the NY-ESO antigen complexed with HLA-A2 with an affinity (*K*_D_ = 7.2 μM) more typical of pMHC/TCR interactions (Chen et al., 2005). Cell lines were generated that expressed the TCR at wild-type (WT) levels, i.e., comparable to Jurkat cells (GPa3b17: 11,262 ± 1,532 TCRs/cell; 1G4: 17,141 ± 5,616 TCRs/cell) and low (Lo) levels (GPa3b17: 236 ± 181 TCRs/cell; 1G4: 488 ± 252 TCRs/cell). The cells were allowed to form contacts with nickelated SLBs presenting gp100/HLA-A2 or 9V/HLA-A2 monomers. Calcium responses were monitored using Fluo-4 AM fluorescence as a readout. Inactivation of ZAP70 and the TCR using CRISPR-Cas9 confirmed that the responses were TCR dependent. Data are presented as means ± SD, n = 3–4 biological replicates; the example shown is representative of two experiments. (B) Tests of CD69 upregulation induced by GPa3b17 TCR signaling. Jurkat T cells expressing wild-type amounts of GPa3b17 TCRs were incubated overnight with wild-type THP-1 cells and THP-1 cells whose expression of β_2_-microglobulin was prevented using CRISPR-Cas9, in the presence and absence of 100 μM gp100 peptide. CD69 expression was analyzed by flow cytometry. Data are presented as means ± SEM, n = 3 biological replicates; the example shown is representative of two experiments. Notably, while signaling was peptide- and pMHC-dependent (p < 0.05, Student’s t test), the GPa3b17 TCR was also triggered in the absence of the gp100 peptide. (C) Schematic showing the design of the expression constructs. DNA sequences encoding GPa3b17 TCR-αβ and CD3 proteins including a GFP2-tagged CD3-δ chain were cloned into three lentiviral expression vectors used to stably transduce CHO cells. The lentiviral constructs, named after the subunits they encoded, were called αγεζ, βεζ, and δ-GFP2. Where necessary, viral 2A sequences (blue arrows) were used to allow expression of multiple proteins by a single virus. Kozak sequences and stop codons are indicated by green and red circles. (D) SDS-PAGE analysis of purified gp100/HLA-A2/TCR, gp100/HLA-A2/TCR/UCHT1 (1/1/2 equiv), and UCHT1 Fab (2 equiv) under reducing (R, left) and non-reducing conditions (NR, right). (E) Binding of UCHT1 Fab to gp100/HLA-A2/TCR assessed by blue native PAGE. The gp100/HLA-A2/TCR was mixed with UCHT1 Fab at the indicated molar ratios prior to loading on the gel. (F) SEC analysis of the TCR in the absence and presence of UCHT1 Fab (3 equiv). For reference, the black trace shows the SEC trace for 1 equiv of UCHT1 Fab alone. (G) Titration of gp100/HLA-A2/TCR and UCHT1 Fab monitored by SEC, demonstrating that two UCHT1 Fab molecules can be bound per gp100/HLA-A2/TCR complex. (H) Flow cytometric analyses of GFP fluorescence (left), UCHT1-Phycoerythrin (PE, middle), and Alexa Fluor 647-labeled gp100/HLA-A2 (pMHC, right) staining of untransduced CHO cells (black), and CHO cells transduced with the following lentivirus combinations: δ-GFP2/αγεζ/βεζ (green), δ-GFP2/αγε/βεζ (orange), and δ-GFP2/αγε/βε (cyan). The median fluorescence intensities following pMHC staining were 4,555 (no virus), 134,852 (δ-GFP2/αγεζ/βεζ), 143,808 (δ-GFP2/αγε/βεζ), and 115,801 (δ-GFP2/αγε/βε). Expression of a gp100/HLA-A2 binding form of the GPa3b17 TCR was not strictly reliant on CD3-ζ.

To isolate a ligand-bound complex, we utilized an affinity-matured TCR-αβ (GPa3b17) (Liddy, 2013) being developed for immunotherapy, i.e., ImmTACs (Liddy et al., 2012), which binds with high affinity (*K*_D_ = 13 pM) to the melanoma-derived gp100 antigenic peptide (YLEPGPVTV) in complex with the most prevalent human leukocyte antigen (HLA), HLA-A^∗^02:01 (HLA-A2). Expressed at both wild-type (WT) and very low levels in leukemic (Jurkat) T cells, the TCR induced calcium signals comparable with those triggered by a non-affinity-matured TCR (Chen et al., 2005) upon stimulation with gp100/HLA-A2-presenting supported lipid bilayers (SLBs; Figure S1A). Moreover, CD69 expression was upregulated on the GPa3b17-expressing cells following incubations with gp100 peptide-pulsed HLA-A2^+^ THP-1 cells (Figure S1B). This confirmed that the signaling capacity of the GPa3b17 receptor is comparable with that of more typical TCRs. The signaling observed in the absence of the gp100 peptide (Figure S1B) likely reflects the cross-reactivity promoting effects of the affinity-enhancing mutations in the HLA-A2-binding regions of the GPa3b17 TCR. cDNAs encoding the six subunits of the TCR, separated by viral 2A ribosome-skipping sites and distributed across three lentiviral vectors (Figure S1C), were expressed in Chinese hamster ovary (CHO) cells because they assembled more homogeneous complexes than HEK293 cells. The cytosolic domains of the CD3 subunits were truncated to limit heterogeneity and the CD3-δ chain tagged with GFP2 to monitor receptor expression and purification. Although partially assembled complexes could reach the surface of CHO cells, gp100/HLA-A2 complexes bound only to cells transduced by all three lentiviruses.

For structural analysis, surface-expressed TCRs were tagged with soluble gp100/HLA-A2 monomers bearing a C-terminal affinity epitope of the Ca^2+^-dependent anti-Protein C antibody HPC-4 (Rezaie and Esmon, 1995). After cell lysis, the assembled TCR complexes were solubilized with glyco-diosgenin (GDN) and isolated using an HPC-4 immunoaffinity matrix and size-exclusion chromatography (SEC). All TCR subunits were identified using SDS-PAGE (Figure S1D). The gp100/HLA-A2/TCR formed a stable complex of ∼250 kDa detectable by native PAGE, which was super-shifted by binding two CD3-ε-specific UCHT1 Fab fragments (Figure S1E). Monodispersity was confirmed by SEC, insofar as the TCR eluted as a single peak that was quantitatively shifted after incubation with two equivalents of UCHT1 Fab (Figures S1F and S1G).

### Cryo-EM analysis of the pMHC-ligated TCR

**Figure S2:**
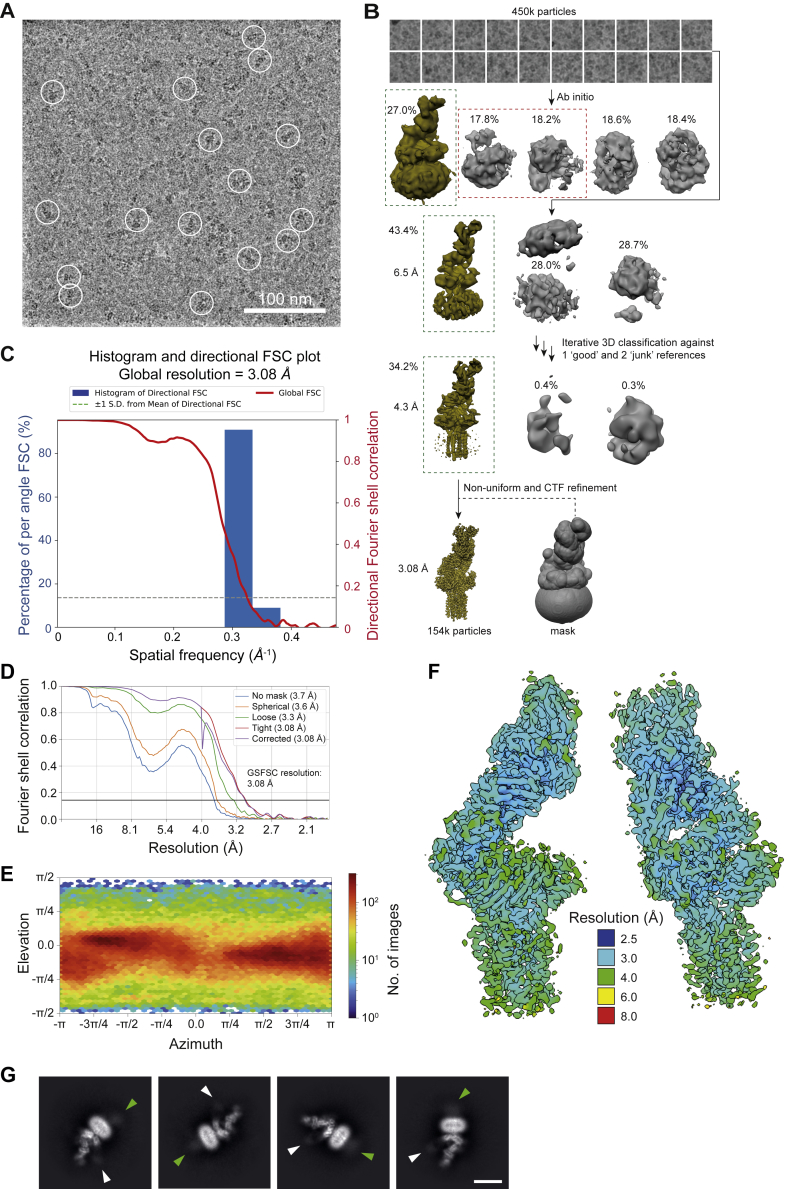
Cryo-EM analysis of the gp100/HLA-A2/TCR complex, related to STAR Methods (A) Typical cryo-EM micrograph on graphene monolayer grids. (B) Cryo-EM data processing workflow. Maps highlighted by green or red dashed boxes served as “good” or “junk” references, respectively, for subsequent rounds of classification/refinement. (C) 3DFSC analysis of the final cryo-EM map (Tan et al., 2017). (D) FSC plot, generated in CryoSPARC (Punjani et al., 2017). The black solid line denotes the FSC = 0.143 cutoff used for resolution determination. (E) Euler angle distribution heatmap for the particles included in the final 3D refinement. The most frequent views are colored in red. (F) Local-resolution estimation computed in CryoSPARC. (G) Reference-free 2D classification of the gp100/HLA-A2/TCR particles. Selected 2D class averages of the gp100/HLA-A2/TCR complex are shown. Green and white arrows indicate densities consistent with GFP2 and UCHT1 Fab, respectively. Scale bar, 100 Å.

**Figure 1:**
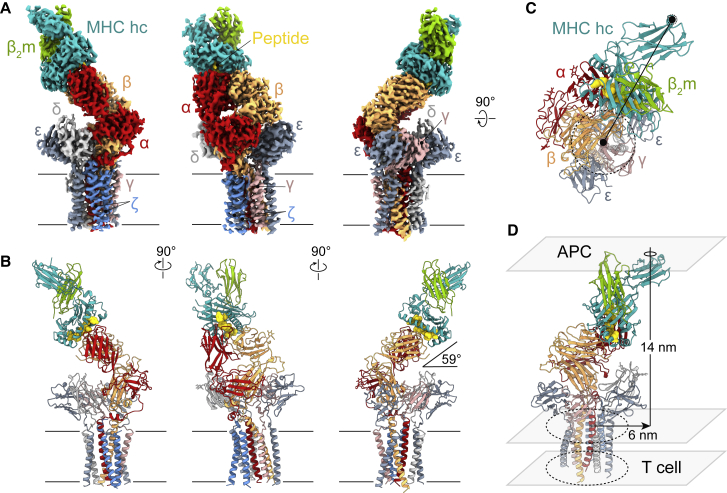
Overall structure of the fully assembled gp100/HLA-A2/TCR complex reveals tilted ligand-binding geometry (A and B) (A) EM density map and (B) atomic model of the fully assembled gp100/HLA-A2/TCR complex viewed parallel to the membrane plane. In (B), protein subunits are depicted in ribbon representation, the tumor-associated gp100 peptide antigen is shown as a space-filling model, and the N-acetylglucosamine moieties are represented by sticks. In (A) and (B), unique polypeptides are individually color-coded, and membrane boundaries are indicated by black lines. The 59° tilt between the gp100/HLA-A2 and TCR extracellular domains and the plasma membrane is denoted. (C) The gp100/HLA-A2/TCR structure viewed from the extracellular space along the membrane normal. Positions of TCR and pMHC transmembrane (TM) anchoring regions are each indicated by black dashed lines with filled black circles at the respective centers. (D) Inter-membrane distance between T cell and antigen-presenting cell (APC), and lateral displacement between the TM centers of TCR and pMHC, as determined from the gp100/HLA-A2/TCR structure. See also Figure S4.

The structure of the gp100/HLA-A2/TCR complex was determined by cryo-EM using all-gold supports covered with a hydrophilized graphene monolayer to preserve the fully assembled multiprotein complex (Figures S2E and S2F). UCHT1 Fab was used to increase particle stability and boost resolution (see online methods STAR Methods). Unexpectedly, almost no density was observed for UCHT1 Fab in the final high-resolution cryo-EM reconstruction, although reference-free 2D classification of the gp100/HLA-A2/TCR particles revealed weak density for the bound Fab and for the GFP2-tag fused to the CD3-δ chain (Figure S2G). An overall resolution of 3.08 Å was obtained for the gp100/HLA-A2/TCR complex, which comprised 11 individual proteins and peptides. The TCR-αβ heterodimer, bound to the trimeric pMHC I, was positioned at the center of the assembly, resting in a “half cup” formed by the heterodimeric CD3-εδ and CD3-εγ ectodomain pairs and supported by the packing of eight transmembrane (TM) helices including the CD3-ζζ TM regions (Figures 1A–1C). The pMHC I-engaged TCR was tilted 59° relative to the plane of the membrane, resulting in an offset of ∼60 Å in the membrane-anchoring points of the TCR and pMHC I (Figures 1C and 1D).

### pMHC recognition by the fully assembled TCR

**Figure 2:**
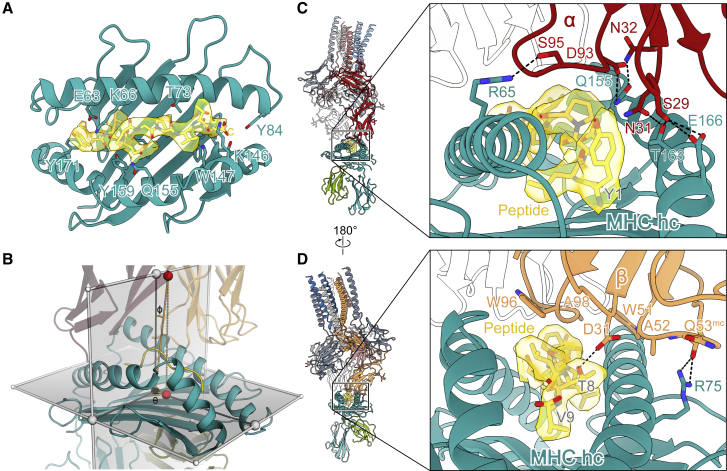
Molecular recognition of tumor-associated gp100/HLA-A2 by the TCR (A) Melanoma antigen gp100 (YLEPGPVTV, yellow sticks) presented in the peptide-binding groove of the MHC I heavy chain (teal ribbon). Peptide-coordinating residues of the MHC I are shown in stick format. The peptide region of the cryo-EM map is depicted as a transparent yellow surface. (B) Geometry of TCR binding to gp100/HLA-A2, characterized in a spherical coordinate system by a rotation angle (Θ) of 93°, a tilt angle (Φ) of 7°, and a distance between the TCR Vα/Vβ center of mass (upper red sphere) and its projection on the horizontal plane (lower red sphere) of 27 Å (dotted line; Singh et al., 2020). (C) Polar interactions (dashed black lines) between residues of TCR-α and gp100/HLA-A2. TCR-β is shown as a colorless cartoon. (D) Details of the interface between TCR-β and gp100/HLA-A2. Hydrogen bonds are depicted as dashed black lines. TCR-α is shown as a colorless cartoon. See also Figure S3.

**Figure S3:**
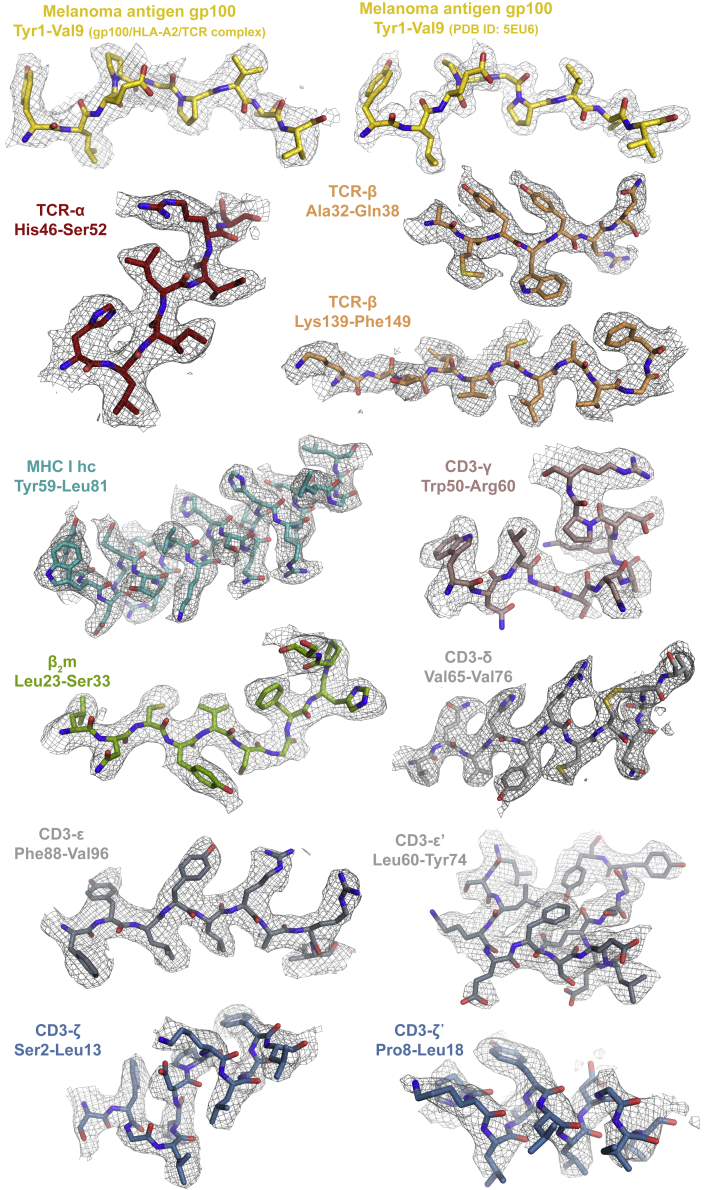
Extracted exemplary regions for each polypeptide chain in the cryo-EM map of the gp100/HLA-A2/TCR complex, related to Figures 1 and 2 In the top row, the cryo-EM map of the fully assembled gp100/HLA-A2/TCR complex at a local resolution of ∼2.6 Å (this work, left) is compared with the electron density for the complex of the soluble PMEL17 TCR-αβ ectodomain bound to gp100/HLA-A2 at 2.0 Å (PDB: 5EU6, right; Bianchi et al., 2016), in the region of the bound heteroclitic peptide.

Having pMHC I bound to the fully assembled TCR revealed how the TM receptor engages ligands. Recognition of pMHC I by the TCR exclusively involved the TCR-αβ complementarity determining regions (CDRs), consistent with soluble TCR-αβ/pMHC ectodomain-only crystal structures (Rudolph et al., 2006). The tumor-associated gp100 epitope was embedded in the MHC I heavy chain in the canonical mode for 9mer peptides, and the quality of the EM map at a local resolution of 2.6 Å allowed unambiguous placement of all residues of the tumor-associated antigen, re-emphasizing the important role of Val9 in anchoring the heteroclitic YLEPGPVTV peptide in the MHC I α1α2 groove (Figures 2A and 2B; Figure S3; Bianchi et al., 2016). The GPa3b17 TCR engaged the pMHC in a diagonal arrangement (rotation: 93°; tilt: 7°) that completely enveloped the peptide and left the pMHC unchanged relative to the unliganded state. The pMHC/TCR interface had a shape complementarity (Sc) (Lawrence and Colman, 1993) of 0.648 and comprised more than twenty contacts, burying an overall surface of 2,154 Å^2^, with the buried area evenly distributed between both TCR-αβ subunits (TCR-α: 49%, TCR-β: 51%). Similar interfaces were found across diverse, soluble pMHC/TCR-αβ ectodomain pairs studied by X-ray crystallography (Rudolph et al., 2006), suggesting that our findings are generalizable. The GPa3b17 TCR had been mutated in three blocks using a “CDR walking” strategy to maximize its therapeutic activity by increasing its affinity (Liddy, 2013). Except for the D95S mutation, which interacts with Arg65 of the MHC I heavy chain, other residues mutated in CDR3α (L98M and V99Q) did not contact the pMHC and must act indirectly, whereas changes to CDR2β (Q51W, I52A, V53Q, and N54G) and CDR3β (I96W and G98A) would have enhanced Sc in the region of the α1 helix of the MHC molecule and/or peptide (Figures 2C and 2D). The improvements in MHC recognition obtained with these mutations likely account for the cross-reactivity of this TCR in our assays (Figure S1B).

### TCR-αβ and CD3 heterodimer stabilization by their connecting peptides

Our high-resolution reconstruction allowed the molecular contacts underpinning the assembly of the TCR to be visualized at a high level of detail. The structures of the extracellular IgSF domains of the TCR-αβ and CD3 heterodimers are well characterized by NMR and X-ray crystallography (Arnett et al., 2004; Garboczi et al., 1996; Garcia et al., 1996; Kjer-Nielsen et al., 2004; Sun et al., 2001). How these parts of each subunit are linked to the membrane by the connecting peptides (CPs) and how the heterodimers interact with one another and with the CD3-ζ homodimer to form the assembled complex has been intensely studied. Although the CP of TCR-α is unusually long (20 residues), the CP of TCR-β is significantly shorter (8 residues); the CPs of the CD3 heterodimers are even shorter and characterized by highly conserved CXXC motifs.

**Figure 3:**
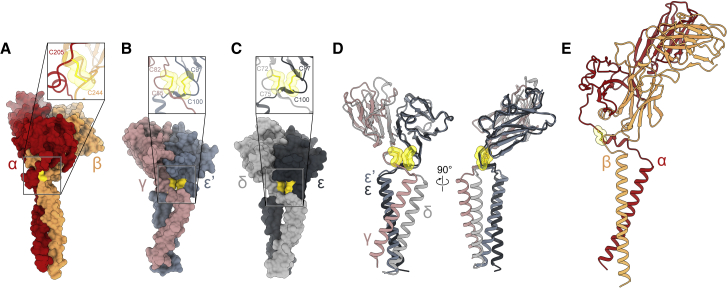
Stabilization of the TCR-αβ and CD3 heterodimer linker regions by the connecting peptides (A) The linker region of the TCR-αβ heterodimer. The TCR-α CP helix fills the space underneath the Cβ domain, stabilizing the linker region. The close-up view shows the intermolecular disulfide bridge (yellow), securing the position of the CP helix. The cystine is represented as sticks, with the atomic van der Waals radii as transparent spheres. (B and C) The rigidity of the CD3-ε′γ (B) and CD3-εδ (C) heterodimers is reinforced by the tight packing of their connecting peptide regions, and most notably by a pair of interacting cystines. The close-up views show the cystines as sticks, with their atomic van der Waals radii as transparent spheres. Chemically identical subunits at different positions in the TCR assembly are distinguished by the prime symbol. (D) Superimposing CD3-ε′γ and CD3-εδ on CD3-ε′ and CD3-ε reveals the remarkably similar architecture of the two heterodimeric CD3 assemblies as putative docking modules. (E) The marked tilt in the ectodomain of the TCR-αβ heterodimer imposed by the linker region, for comparison with similarly tilted ectodomains of the CD3-ε′γ and CD3-εδ heterodimers in (D). The topologies of the heterodimers likely facilitate ectodomain docking and the simultaneous close association of their TM regions.

For the TCR-αβ and CD3 heterodimers, the CPs serve to create rigid links to the membrane, but in two quite different ways. For TCR-αβ, the TCR-α CP reaches around and under TCR-β, where it forms a short helix that fills the space between TCR-Cβ and the tops of the TCR-αβ TM helices. The TCR-α CP helix is held in place by a disulfide bond to the TCR-β CP, bringing the CPs into proximity and reinforcing the linker region (Figure 3A). In the CD3 heterodimers, an elegant and evidently highly conserved arrangement of disulfides plays the major stabilizing role in the linker region. Strikingly, the cysteines comprising the conserved CXXC motifs adjacent to the TM regions of the CD3-δ, -ε, and -γ subunits form two pairs of interacting intramolecular cystines in each heterodimer (Figures 3B and 3C) which, in the assembled complex, are located either side of the “top” of the TCR-αβ TM bundle. The cystines are stabilized in this arrangement by hydrophobic interactions, accounting for their remarkable resistance to reducing agents (Brazin et al., 2014). For each CD3 heterodimer, the paired cystines dramatically redirect the CPs, forcing them to “cross-over” (Figure 3D). This in turn separates and re-positions the TM regions of each subunit under the other subunit in a reciprocal arrangement. Perhaps, more importantly, by securing the positions of the CPs, the cystine pairs rigidify the linker regions of each heterodimeric subunit. Superpositions reveal the remarkable similarity of the two CD3 heterodimers as structural modules (Figure 3D), which can be thought of as semi-autonomous assemblies stabilized by their CPs that assemble with TCR-αβ heterodimers via interactions across three layers, extending from the extracellular space to the membrane (discussed below). Notably, the CPs allow the ectodomains of all three heterodimers to tilt back or forward relative to the long axis of their TM regions, likely facilitating close TM packing (Figures 3D and 3E).

### Quaternary interactions of the dimeric TCR-αβ, CD3-εδ, -εγ, and -ζζ ectodomains

**Figure 4:**
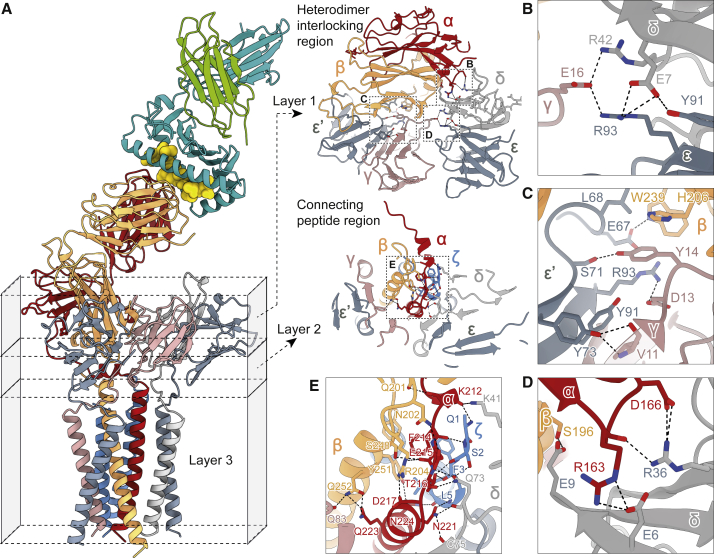
Conformational stabilization of the TCR through a network of interlocking, multivalent interactions (A) From the constant regions of TCR-αβ to the membrane, the TCR inter-subunit contacts can be divided into three layers: the folded ectodomains (layer 1), the connecting peptide region (layer 2), and the TM domains (layer 3). (B–D) Close-up views of interactions in layer 1. (E) Close-up view of the interaction network in the connecting peptide region (layer 2). Hydrogen bonds and salt bridges in (B)–(E) are depicted by black dashed lines. See also Figure S4A.

**Figure S4:**
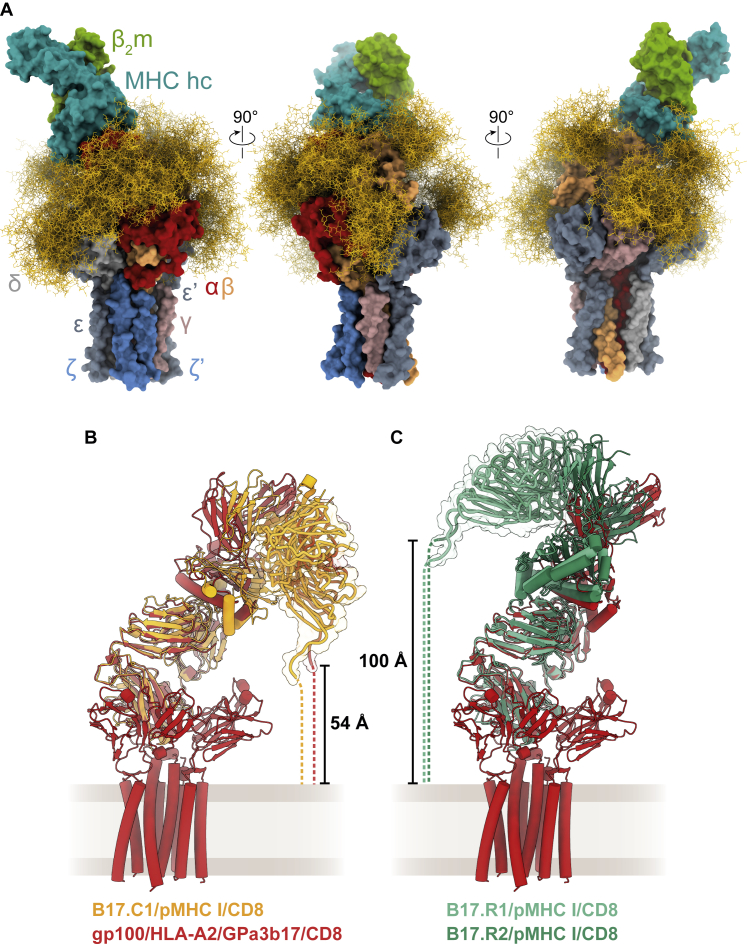
Glycosylation sites of the liganded TCR and influence of the pMHC I/TCR docking polarity on the CD8-binding sites, related to Figures 2 and 4 (A) The different subunits of the gp100/HLA-A2-bound GPa3b17 TCR are shown with their solvent-excluded surfaces. N-linked glycans ((NeuNAc-Gal-GlcNAc)_2_Man_3_(Fuc)(GlcNAc)_2_) were modeled using the molecular dynamics (MD) software pipeline GlycoSHIELD (Gecht et al., 2022) and are depicted as tan-colored sticks. For each glycosylation site, 50 conformers are shown. The cryo-EM map contained clear “density” only for the Asn-linked GlcNAc moieties, suggesting that N-glycans are not involved in any protein interfaces. (B) The canonical TCR/pMHC I docking polarity, as exemplified by the structures of B17.C1/pMHC I (PDB: 7JWJ) and gp100/HLA-A2/GPa3b17 TCR complexes, places the binding site of the membrane-anchored CD8 in a position that is more accessible than in the reversed TCR/pMHC I docking polarity (C), as observed for B17.R1/pMHC I (PDB: 5SWZ) and B17.R2/pMHC I (PDB: 7JWI). The CD8αβ heterodimers (PDB: 3DMM) are depicted as tubes with their surfaces as transparent envelopes. The distances that must be traversed between the C terminus of the CD8β subunit and the membrane are shown as dashed lines.

The tilted geometry of each heterodimeric subunit imposed by the rigid linkers enabled substantial interactions across three structural layers for each subunit pair. In the region of the IgSF domains (layer 1, Figure 4A), CD3-εδ and CD3-εγ were held together by small interfaces comprising just a few residues. This included a three-way interaction close to TCR-αβ wherein a glutamate in CD3-γ (Glu16) slotted between arginines in CD3-δ (Arg42) and CD3-ε (Arg93, supported by a salt- and H-bridge network involving Glu7 of CD3-δ and Tyr91 of CD3-ε, respectively; Figure 4B). The CD3-εγ and CD3-εδ heterodimers were each in turn anchored to the base of the TCR-αβ constant regions: on one side by a contact that had, at its core, a four-way hydrophobic interaction between His206 and Trp239 of TCR-β, Tyr14 of CD3-γ, and Leu68 of CD3-ε′ (Figure 4C), in the center by Ser196 of TCR-β contacting Glu9 of CD3-δ, and, on the other side, by a network of reciprocal side- and main-chain contacts between Arg163 and Asp166 of TCR-α and Glu6 and Arg36 of CD3-δ (Figure 4D). These highly directional interactions positioned CD3-εγ adjacent to TCR-β, and CD3-εδ next to TCR-α, in this way stabilizing the tilted arrangement of TCR-αβ relative to the membrane. N-glycans attached to the TCR are unlikely to have a direct impact on complex assembly. The cryo-EM map yielded “density” only for the Asn-linked GlcNAc moieties, suggesting that N-glycans are not involved in any protein interfaces (Figure S4A).

Interactions in the linker region (layer 2; Figure 4A) were dominated by TCR-α and involved CD3-ζζ. TCR-α formed three main contacts with the disulfide-linked CD3-ζ dimer (Figure 4E). First, the backbone of Lys212 of TCR-α contacted the N terminus of CD3-ζ. Second, Phe214 of TCR-α formed a main-chain contact with Ser2 of CD3-ζ and a hydrophobic cluster with Phe3 and Leu6 of CD3-ζ as well as Phe246 and Tyr251 of TCR-β. Third, Thr216 and Asn221 of TCR-α bound main-chain atoms of Ser2 and Phe3, as well as Leu5 of CD3-ζ, respectively. In addition, Asn221 of TCR-α established main-chain contacts with Gln73 and Cys75 of CD3-δ (Figure 4E). The side chain of Gln73 also interacted with main-chain atoms of Thr216 in TCR-α. In stark contrast, interactions of TCR-β with the CD3 subunits in this region were limited to just one hydrogen bond with the main chain of Gln83 in CD3-γ, apart from the participation of Phe246 (TCR-β) in the hydrophobic cluster.

### Transmembrane assembly including the contribution of sterol lipid

**Figure 5:**
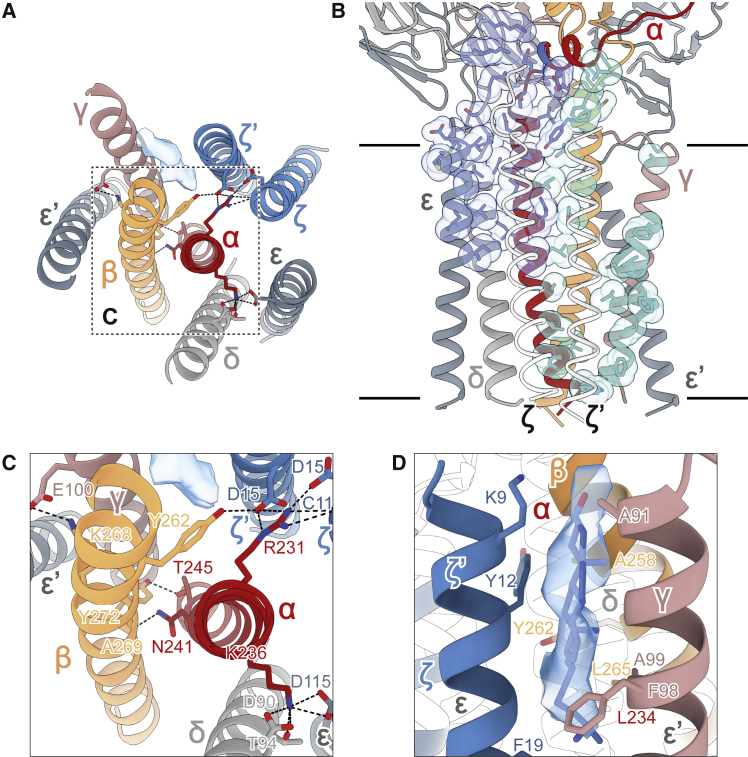
Transmembrane assembly including the contribution of sterol lipid (A) Extracellular view of the transmembrane domain (layer 3). (B) The two CD3-ζ chains (shown in white) are positioned in very different environments within the CD3 TM assembly. The view is related to Figure 4A by a 165° rotation around the longest axis of the complex. Residues interacting with CD3-ζ (blue) and CD3-ζ′ (cyan) were identified using the program PISA (Krissinel and Henrick, 2007) and are shown as sticks and with their atomic van der Waals radii as transparent spheres. (C) Tight associations in the outer leaflet portions of the TCR-α and TCR-β TMs with CD3-εδ and CD3-ε′γ, respectively, enable the intra-membrane neutralization of positive charges in TCR-α and TCR-β. Hydrogen bonds and salt bridges in (A) and (C) are depicted by black dashed lines. (D) Extra non-protein density (transparent blue surface) was observed in the outer leaflet between the TM helices of CD3-ζ′ and CD3-γ, likely matching a cholesterol molecule.

The interlocking arrangements thus established in the folded ectodomain and linker regions were reinforced by interactions within the membrane (layer 3; Figure 4A). As in the unliganded TCR (Dong et al., 2019), the TMs of the CD3 dimers packed around a centrally located TCR-αβ TM helical bundle spanning the length of the membrane because interactions in the linker region had placed the CD3-γε TM next to the TCR-β TM, and CD3-δε alongside the TCR-α TM (Figure 5A). However, we observed a pronounced asymmetry in the degree of interdigitation of the TM helices across the membrane (Figure 5B). Close associations within the outer, but not the inner, leaflet of TCR-α and TCR-β TMs with CD3-εδ and CD3-εγ, respectively, favored the well-characterized intra-membrane neutralization of the positive charges of Lys236 of TCR-α (by Asp115 of CD3-ε as well as Asp90 and Thr94 of CD3-δ), Arg231 of TCR-α (by Asp15 of each CD3-ζ chain and main-chain atoms of Cys11 of CD3-ζ), and Lys268 of TCR-β (by Glu100 of CD3-γ; Figure 5C; Call et al., 2004). Alongside the prominent salt bridges in the TM regions, two tyrosine residues provide long-range connectivity, involving Tyr262 of TCR-β with Asp15 of CD3-ζ′, and Tyr272 of TCR-β with Thr245 of TCR-α.

Intra-membrane associations of the CD3-ζ homodimer complete the assembly. By packing against TCR-α, CD3-ζζ allowed neutralization of Arg231 of the α chain (by Asp15 of each CD3-ζ chain) but positioned the two CD3-ζ chains in very different environments (Figure 5B). One CD3-ζ polypeptide was displaced from the rest of the TM assembly, especially in the cytosolic leaflet, where it was likely surrounded by unstructured lipids, reminiscent of intra-membrane proteases (Sun et al., 2016). The other CD3-ζ chain formed very few protein-protein contacts at all. Instead, unexpectedly, extra density was seen in the outer leaflet of the membrane sandwiched between the TMs of CD3-ζ and CD3-γ, which we assigned to cholesterol (Figure 5D). Notably, although production of the complex for structural analysis required all three expression plasmids, we observed that pMHC-binding TCR complexes formed readily in CHO cells after we specifically deleted CD3-ζ (Figure S1H), suggesting that ζ-chain homodimer incorporation comprises a late step in TCR assembly. This is in marked contrast to normal T cells as well as T cell hybridomas and leukemic T cell lines, which express very low levels of TCR in the absence of CD3-ζ (Geisler et al., 1989; Lin et al., 1997; Sussman et al., 1988). Evidently, receptor editing is far more stringent in T cells than in CHO cells. Sterol lipids, intercalated among the TM regions, may nevertheless have an important role in stabilizing a TCR-αβ/CD3-δγε_2_ intermediate while also contributing to a composite docking site for the ζ-chain homodimer.

### pMHC-induced changes in the fully assembled TCR

**Figure 6:**
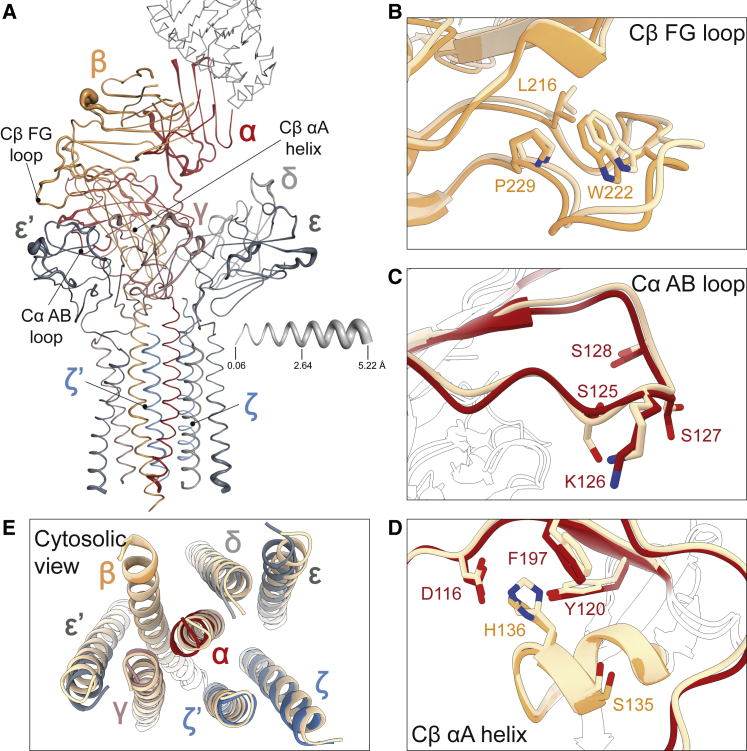
Structural rigidity of the TCR membrane complex (A) Structural differences between the gp100/HLA-A2-bound GPa3b17 TCR and unliganded complex (Dong et al., 2019) mapped onto the gp100/HLA-A2-bound TCR. Thickness of the putty cartoon representation corresponds to the distance between equivalent Cα atoms after superposition of the two structures. Distances vary between 0.06 and 5.22 Å. The gp100/HLA-A2 structure is shown as gray ribbon. (B–E) Structural differences between gp100/HLA-A2-bound and unliganded (light wheat color) TCR in regions previously postulated to be sites of pMHC-induced allosteric regulation, including the Cβ FG loop (B), Cα AB loop (C), Cβ αA helix (D), and the C termini of TM helices (E). Selected residues are shown as sticks. See also Figure S5, Figure S6, Figure S7.

**Figure S5:**
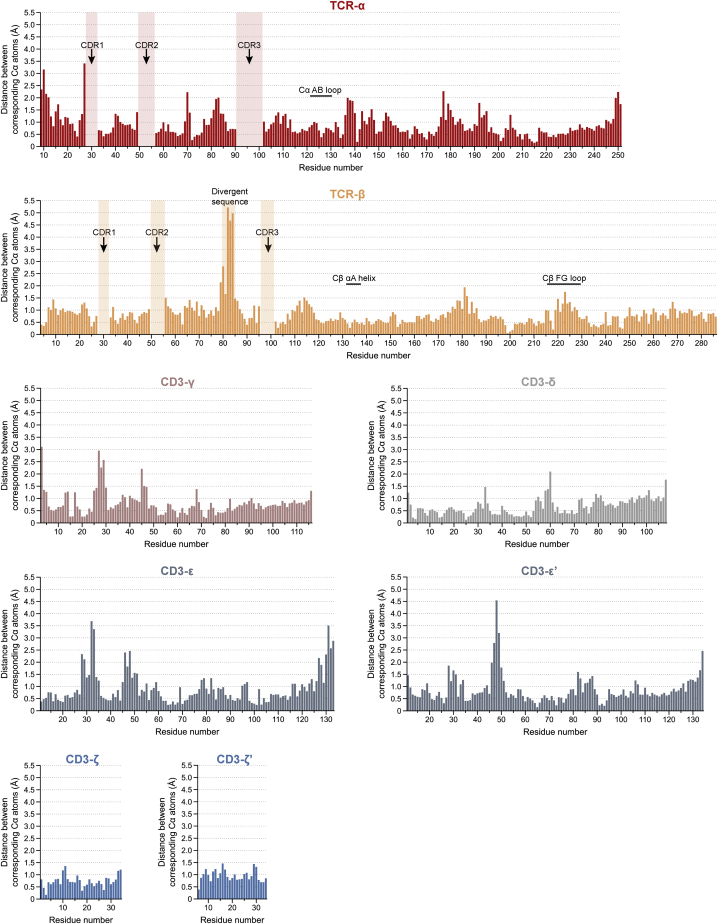
Structural comparison of gp100/HLA-A2-bound and unliganded TCR, related to Figure 6 The structures of gp100/HLA-A2-bound GPa3b17 TCR and the unliganded receptor (Dong et al., 2019) were superimposed, and the Cα atomic distance for each residue in all subunits was calculated. Notable regions previously implicated in allosteric mechanisms of signaling are marked. Note that the highly divergent CDR motifs were not included in the analysis and are indicated by pale-colored rectangles.

Our structure of the pMHC/TCR complex allowed us to examine the effects of pMHC binding on the fully assembled receptor. Comparing this structure with the backbone conformation of the cryo-EM reconstruction at 3.7 Å of the unliganded TCR (Dong et al., 2019) indicated that the TCR is remarkably resistant to any form of ligand-induced structural rearrangement. The root-mean-square-deviation (RMSD) between the two structures over all Cα atoms was 1.2 Å (Figures 6A and S5). Notably, although crystallographic and NMR studies had suggested that pMHC binding induces changes in the region of the TCR-Cβ FG loop (Rangarajan et al., 2018; Figure 6B), the TCR-Cα AB loop (Beddoe et al., 2009; Rangarajan et al., 2018; Figure 6C) and the TCR-Cβ αA helix (Natarajan et al., 2017; Rangarajan et al., 2018; Figure 6D), these regions were essentially identical, exhibiting Cα-RMSDs of 0.7, 0.5, and 1.0 Å, respectively. Moreover, despite large differences in construct design and sample preparation, there were only minor displacements of the C termini of the membrane-spanning regions, with the largest being observed for the tips of peripherally located CD3-ε (3.5 Å for Cα-Lys131) and CD3-ε′ (2.5 Å for Cα-Lys134; Figure 6E).

### Comparison of high-affinity and wild-type pMHC/TCR complexes by MD simulation

The GPa3b17 TCR binds with high affinity to the melanoma-derived gp100/HLA-A2 complex (*K*_D_ = 13 pM). As such, it was theoretically possible that the high affinity of the interaction “traps” functionally relevant dynamics that are important for signaling in response to typical, lower-affinity ligands (*K*_D_ = 1−100 μM) (Davis et al., 1998). To investigate this we used *in silico* mutagenesis to return the GPa3b17 TCR to the parental “WT” gp100 TCR sequence, which binds gp100/HLA-A2 with an affinity of *K*_D_ = 22 μM (Liddy, 2013) (mutated residues in TCR-αβ are listed in the STAR Methods section). We then performed 4 μs-long atomistic MD simulations of gp100/HLA-A2 in complex with partial (i.e., TCR-αβ) and fully assembled models of the GPa3b17 and WT TCRs, respectively (see STAR Methods). The fully assembled simulation systems were embedded in a realistic model of the plasma membrane.

**Figure S6:**
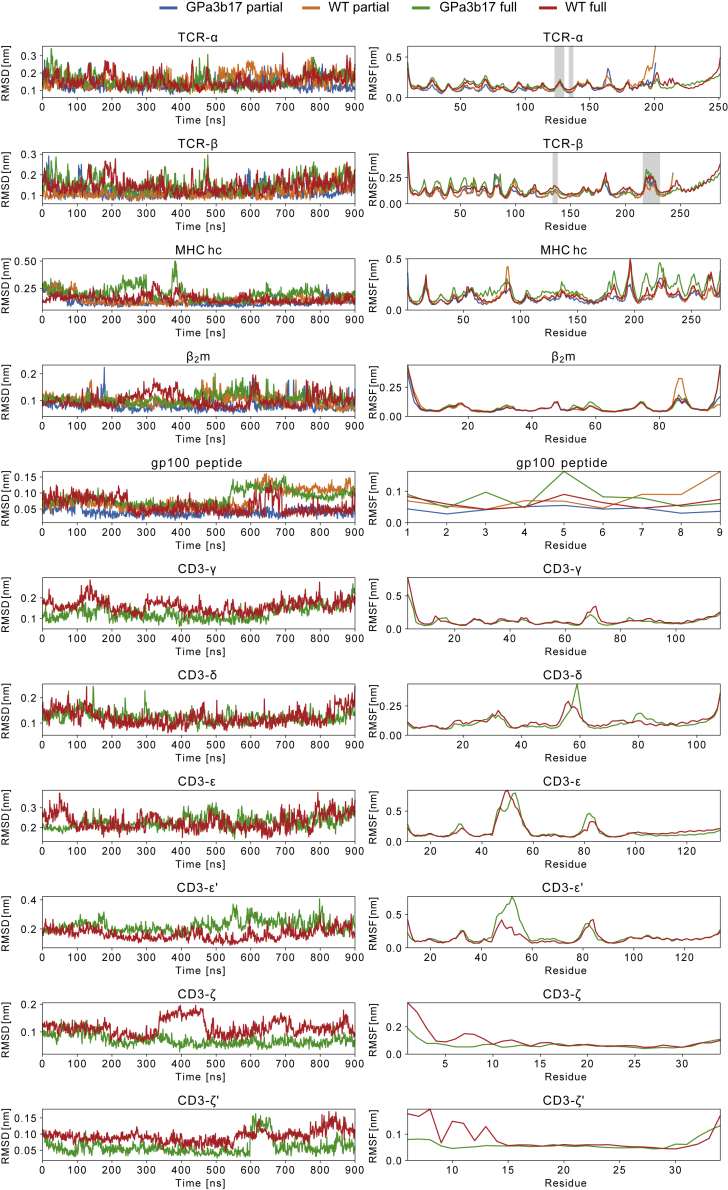
Comparison of the GPa3b17 and parental WT TCR complexes, related to Figure 6 RMSD (left) and RMSF (right) values of the individual subunits with respect to their mean structure are reported for the four simulation systems: gp100/HLA-A2-bound GPa3b17 TCR comprising the soluble TCR-αβ subunits (“GPa3b17 partial,” blue lines), gp100/HLA-A2-bound WT TCR comprising the soluble TCR-αβ subunits (“WT partial,” orange lines), gp100/HLA-A2-bound GPa3b17 fully assembled TCR (“GPa3b17 full,” green lines), and gp100/HLA-A2-bound WT fully assembled TCR (“WT full,” red lines). Residues of the Cβ FG loop (residues 216–231), Cα AB loop (residues 134–137), and around the Cβ αA helix (residues 134–137 of TCR-α and 134–138 of TCR-β) are shaded in gray.

**Figure S7:**
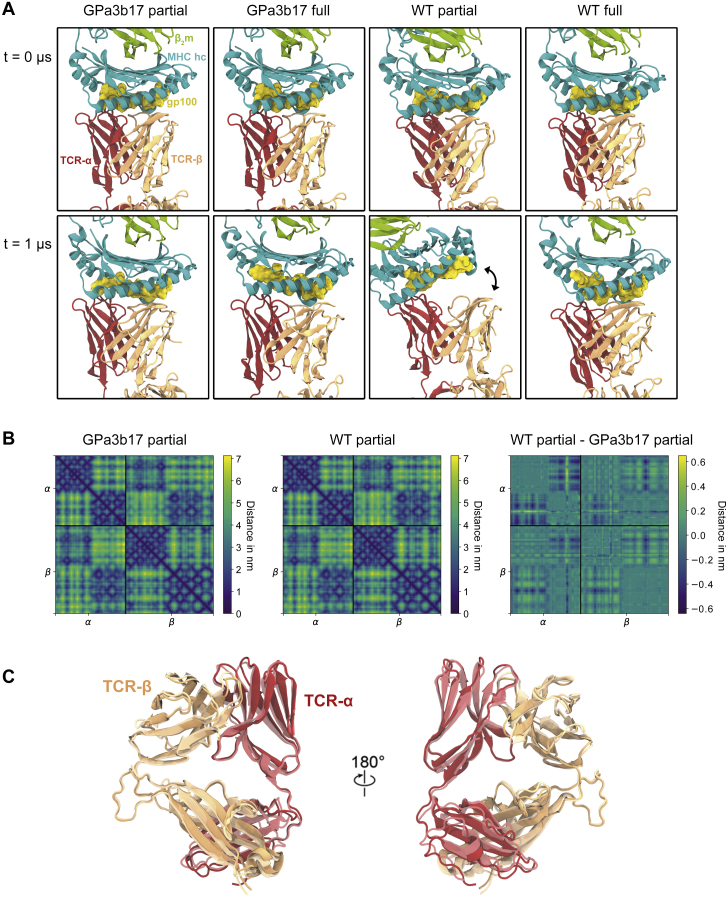
Gp100/pMHC/TCR-αβ interface, TCR-αβ distance matrices, and mean TCR structures from simulation, related to Figure 6 (A) Renders of first (t = 0 μs) and last (t = 1 μs) simulation snapshots of the gp100/HLA-A2/TCR-αβ interface from simulations of the partial and fully assembled GPa3b17 (left) and WT (right) TCRs. (B) Distance matrices reported for mean TCR-αβ simulation structures of partial GPa3b17 and WT TCRs bound to gp100/HLA-A2. The distance map (WT partial − GPa3b17 partial) reveals that only subtle changes in the internal organization of TCR-αβ are associated with the GPa3b17 mutations. (C) Renders of the partial (TCR-αβ) mean simulation structures. Darker colors identify the gp100/HLA-A2-bound partial GPa3b17 TCR, and lighter colors the gp100/HLA-A2-bound partial WT TCR. The proteins were rendered with visual molecular dynamics (VMD) v1.9.3 (Humphrey et al., 1996).

RMSDs and root-mean-square fluctuations of the complex subunits revealed that the subdomains of the GPa3b17 and WT TCR complexes were stable throughout the simulations (Figure S6), with two local exceptions: one of the two CD3-ζ TM helices unraveled slightly in both fully assembled TCR simulations, possibly owing to truncations of the simulation models at the N termini of the CD3-ζ cytosolic regions, and the CD3-ε and CD3-ε′ D β strands (residues 52–56) exhibited a tendency to dislodge from their respective β sheets in the fully assembled TCR simulations (Video S1). We hypothesize that the binding of UCHT1 Fab to the preceding loop has a stabilizing effect on this region that is not captured in our simulation model without the Fab. As expected, the simulations showed that the interaction of gp100/HLA-A2 with the WT TCR was more transient than the interaction of gp100/HLA-A2 with GPa3b17. Both the partial and fully assembled GPa3b17 TCRs maintained stable interactions with gp100/HLA-A2 (Figure S7A). In contrast, only the fully assembled WT TCR remained stably bound to gp100/HLA-A2, with the pMHC interaction of the partial WT TCR loosening during the simulation. However, both the GPa3b17 and WT TCR-αβ subdomains exhibited similar internal dynamics and mean simulation structures throughout, with near-identical distance matrices of TCR-αβ residue pairs (Figures S7B and S7C). Likewise, the TM domains of the complex exhibited similar dynamics in the simulations of the fully assembled GPa3b17 and parental TCRs (Figure S6). Therefore, the enhanced affinity of the GPa3b17 receptor was not associated with marked changes in the internal stability or dynamics of the ligand-bound complex. These data indicate that lower-affinity TCRs also resist ligand-induced structural rearrangements.

Video S1. Atomistic molecular dynamics simulation, related to Figures 6, S6, and S7 The gp100/HLA-A2-bound fully assembled GPa3b17 TCR was embedded in a model of a plasma membrane (upper leaflet: POPC 33.3 mol%, PSM 33.3 mol%, cholesterol 33.3 mol%; lower leaflet: POPC 35 mol%, POPE 25 mol%, POPS 20 mol%, cholesterol 20 mol%) for 900 ns simulation time. The subunit coloring follows that of the main figures. The plasma membrane atoms are shown as transparent green points, with phosphate atoms highlighted by solid spheres to delineate the membrane bounds. Water and ions are not shown for clarity. The movie was rendered with Visual Molecular Dynamics (VMD) v.1.9.3 (Humphrey et al., 1996).

### Implications for co-receptor interactions at the membrane

**Figure 7:**
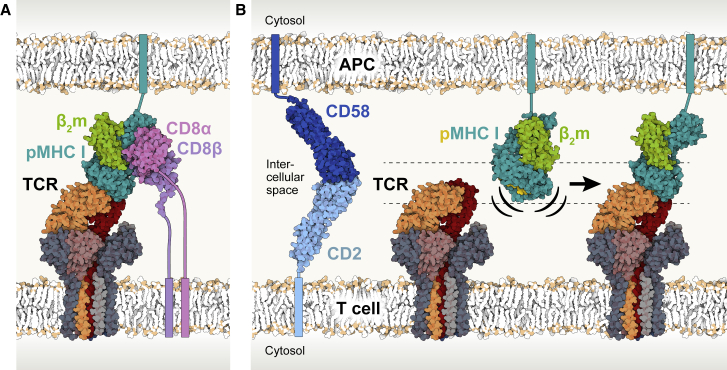
Overall geometry of the TCR membrane complex and implications for co-receptor binding and antigen recognition (A) Following pMHC binding, the co-receptor CD8αβ can readily dock onto the α3 domain of the MHC heavy chain to augment TCR signaling. (B) Productive pMHC/TCR binding requires membrane proximity, which is facilitated by CD2 and CD58 adhesion molecules on the T cell and APC, respectively. Rigid-body motion of the pMHC suffices to enable TCR binding, leading to signaling. See also Figures S4B and S4C.

Finally, by docking CD8 to pMHC in the liganded structure, in the manner of the complex formed by the soluble truncated ectodomains of murine H-2D^d^ and CD8αβ (Wang et al., 2009a), we were able to reconsider the role of co-receptors in signaling by the TCR. We found that CD8 binds comfortably to the “underside” of HLA-A2 in the pMHC/TCR complex (Figure 7A). The “locking” of the binding site in such a position by TCR engagement of pMHC would favor associations with any protein that is tethered to the same membrane as the receptor and interacts in *trans* with the apposing cell, such as CD8. This may explain the temporal sequence of events leading to assembly of the ternary pMHC/TCR/CD8 (or CD4) complex, with the coreceptors arriving last (Jiang et al., 2011).

The structure of a TCR that binds to pMHC in an arrangement that is the reverse of what is typically seen (Rudolph et al., 2006) was recently determined (Zareie et al., 2021). This positions CD8 at the “top” of the pMHC rather than underneath, a location that is likely to be less accessible than in the canonical arrangement owing to displacement of the binding site by ∼50 Å (Figures S4B and S4C). Although probably flexible, it seems unlikely that the extended mucin-like regions of CD8 (Merry et al., 2003) easily accommodate binding to pMHC in both positions. CD4, which is comprised of concatenated IgSF domains (Wu et al., 1997), is even less likely to have the necessary length variability. These considerations suggest a second explanation for the failure of the reverse-topology receptor to signal. In addition to the inability of CD8 to deliver kinases to the cytosolic CD3 chains in this arrangement (Zareie et al., 2021), signaling could be prevented by the inability of CD8 to dock efficiently with the preformed pMHC/TCR complex, owing to the unique geometry of the complex.

## Discussion

We determined the structure of a fully assembled TCR in complex with a cognate pMHC ligand. Because we could avoid chemical cross-linking, our data reveal that a pMHC-bound TCR is remarkably stable. We provide a near-atomic view of how the TCR achieves this conformational stability, which involves (1) asymmetric, multivalent contacts between the CD3-εδ and CD3-εγ ectodomains as well as the constant regions and CPs of the TCR-αβ subunits consistent with mutational and NMR studies (Fernandes et al., 2012; He et al., 2015; Kuhns et al., 2010; Touma et al., 2007; Wang et al., 2009b), (2) the assembly of the CD3 TMs around a centrally located TCR-αβ TM bundle in an arrangement that is relatively loose but crucially favors charged TM-residue neutralization, and (3) an intercalated sterol lipid that supports packing of the TM bundle in the region of the outer membrane leaflet. Our work emphasizes, especially, the role of conserved, interacting cystines in the CPs of the CD3-εδ and CD3-εγ subunits in rigidifying the two CD3 heterodimers, and the similarity of these heterodimers as structural units. The essential role of the cystines explains the impact of mutating these residues on TCR assembly and signaling, and thymocyte development (Touma et al., 2007; Wang et al., 2009b). To our knowledge, this arrangement of paired, hydrophobically interacting cystines is unique to the TCR.

We speculate that the likely rigid conformations of the CD3-εδ, CD3-εγ, and TCR-αβ subunits facilitate their docking. The structural similarity of the CD3 heterodimers also suggests that in some circumstances, they might exhibit a degree of interchangeability during receptor assembly. We predict γδTCRs and the pre-TCR, each of which can assemble in the absence of CD3-δ (Dave et al., 1997), and the TCRs of chickens, which have just two CD3 genes (encoding ε and a single γ/δ ortholog) (Göbel and Dangy, 2000), to have the same overall arrangement of subunits we observe here. The signaling subunits of the BCR, Ig-α and Ig-β, which lack equivalent cysteine residues in their CPs, likely assemble in a different manner (see, e.g., Radaev et al., 2010). The multivalent nature of the subunit interactions, and our finding that the formation of ligand binding-competent TCRs requires all subunits to be present except CD3-ζ, suggest an initial “all-in or none-in” mode of assembly of the heterodimeric subunits, followed by incorporation of CD3-ζζ. Being largely buried, sterol lipid incorporation into the TCR is different to that observed for G protein-coupled receptors, wherein cholesterol is attached to the outer surface of the TM bundle (Dunne et al., 2009). Cholesterol is proposed to stabilize non-signaling TCRs (Swamy et al., 2016) but might instead have a structural role, consistent with the signaling-inhibitory properties of cholesterol sulfate (Wang et al., 2016a), which could affect complex stability. Our finding that sterol lipid, presumably cholesterol, is intercalated within the TM regions of the liganded GPa3b17 TCR is mirrored by recent work showing cholesterol to be similarly located in an unliganded TCR (Chen et al., 2022). However, it seems highly unlikely that cholesterol would have a gating role in signaling as proposed by Chen et al. (2022) if it is present in both structures.

Most importantly, this initial view of a ligand-bound receptor reveals the underpinnings of TCR function. Comparison with the unliganded structure suggested that except for local changes at the ligand-binding site, the TCR resists perturbation and interacts as a “rigid body” with gp100/HLA-A2. Previously, it was proposed that pMHC binding triggers allosteric rearrangements of the CD3-ζ subunits (Lanz et al., 2021; Lee et al., 2015) or TCR-α and TCR-β constant regions (Beddoe et al., 2009; Natarajan et al., 2017; Rangarajan et al., 2018), but this is not confirmed here. “Dynamic allostery,” i.e., the transmission of signals to distal sites via changes in protein dynamics in the absence of overt structural transitions (Mariuzza et al., 2020), warrants further investigation. Nonetheless, the TCR does not exhibit the intrinsic conformational flexibility needed for allostery-based signaling in the manner of, e.g., G protein-coupled receptors (Duncan et al., 2020). It needs to be acknowledged that these conclusions rest on an analysis of a high-affinity TCR/pMHC complex, although our MD simulations suggest that the receptor’s structural dynamics are not fundamentally altered by more typical, lower-affinity ligand interactions.

Since current cryo-EM technology does not allow the pMHC/TCR complex to be subjected to forces, caution must be exercised with respect to their role in signaling. Here, we note only that theories of mechanotransduction (e.g., Kim et al., 2009) must account for signaling by all ligands. Our structure reveals that pMHC and agonistic antibodies would each “pull” on different parts of the TCR and along different trajectories, making it unlikely that both classes of ligands could trigger a shared sequence of conformational rearrangements. Moreover, forces acting via the TCR were only barely measurable for T cells interacting with fluid-phase SLBs presenting TCR ligands (Göhring et al., 2021). Our finding that untethered pMHC and Fab adducts that only slow the diffusion of the receptor are capable of triggering signaling also suggests that strong forces are unnecessary (Chen et al., 2021). Being essentially unchanged by pMHC binding, the GPa3b17 TCR is also unlikely to exhibit any new tendency to oligomerize after interacting with ligands, as has been suggested (Kuhns et al., 2010). Instead, contrary to general expectation, the TCR adopts a stable structure that appears to be primed for ligand engagement and signaling without an apparent need for spontaneous structural rearrangements. To the best of our knowledge, in this respect, the TCR differs from all other classes of signaling receptors. Overall, the structural data are consistent with the notion that a TCR ligand may need only to bind the receptor, holding the TCR in regions of the membrane that favor signaling (Davis and van der Merwe, 2006). This explains both the intrinsic structural diversity of the TCR (Morrissey et al., 2021) and its ability to be triggered by ligands that engage it in so many profoundly different ways (Sundberg et al., 2002).

The pMHC binding site on the TCR is tilted and located on the “side” of the receptor owing to CD3 locking the TCR-αβ subunits at an angle of 59° relative to the cell surface. Positioning the ligand-binding region in this way would constrain *de novo* “head-to-head” encounters of the TCR- and pMHC-binding sites in a direction orthogonal to the respective cell surfaces. Similarly, a binding site at the “top” of the TCR would hinder binding if the inter-membrane distance was pre-set by similarly sized adhesion proteins such as CD2 and CD58, and only lateral movement was possible. We propose that the important role of CD2 and CD58, or of other similarly sized cell-cell interacting proteins, is to create an initial overlap in the positions of the TCR and pMHC-binding sites (Figure 7B). Along with the remarkably fast two-dimensional pMHC/TCR binding kinetics (Huang et al., 2010), pre-alignment of the membranes by CD2 and CD58 would facilitate antigen scanning by the TCR. In turn, formation of the pMHC/TCR complex could favor co-receptor recruitment. The structural data therefore explain the key role of small adhesion proteins in, e.g., tumor killing by T cells (Frangieh et al., 2021). More importantly, access to the structure of a fully assembled ligand-bound receptor brings us substantially closer to solving the enigma of TCR triggering.

### Limitations of the study

Caveats of our study are (1) that we draw inferences about TCR signaling from a receptor bound to a soluble pMHC ligand, (2) that we removed the cytosolic regions of the complex, and (3) that we used a high-affinity TCR to assemble a pMHC/TCR complex that was stable enough for cryo-EM analysis. Future work is needed to determine whether the TCR is as resistant to structural rearrangements when it is ligated by a membrane tethered ligand as it is when it binds a soluble pMHC. Previous work has demonstrated that the cytosolic regions of an unliganded TCR are unstructured (Dong et al., 2019), and it will be important to determine whether these regions are also unstructured in fully assembled, ligand-bound complexes, ideally in the presence of lipids reflecting the composition of the T cell plasma membrane. Finally, the possibility that all TCR interactions are essentially rigid body in character needs to be tested via the determination of structures of other fully assembled, ligand-bound TCRs, including those with more typical ligand affinities, as soon as this becomes possible.

## STAR★Methods

### Key resources table

**Table:** 

REAGENT or RESOURCE	SOURCE	IDENTIFIER
**Antibodies**		

Anti-CD3ε clone UCHT-1	Hybridoma	PMID 6788570
Anti-CD3ε clone UCHT-1 FITC conjugated	Biolegend	Cat# 300452
Anti-CD3ε clone UCHT-1 PE conjugated	Biolegend	Cat# 300408
Anti-CD8 clone SK1 PE-Cy7 conjugated	Biolegend	Cat# 344750
Anti-CD69 clone FN50 Pacific Blue conjugated	Biolegend	Cat# 310920

**Bacterial and virus strains**		

Rosetta 2 (DE3) pLysS	Novagen	Cat# 71404-3
One Shot Top10 chemically competent *E. coli*	Invitrogen	Cat# C404010

**Chemicals, peptides, and recombinant proteins**		

Alexa Fluor™ 647 Antibody Labelling Kit	Invitrogen	Cat# A20186
Alexa Fluor™ 555 Antibody Labelling Kit	Invitrogen	Cat# A20187
Amicon Ultra-15 centrifugal filters	Merck, Millipore	Cat# UFC901096
Ampicillin	Sigma	Cat# A9518-25G
L-Arginine	Sigma	Cat# A5131-1KG
BD Quantibrite™ Beads PE Fluorescence Quantification Kit	BD Biosciences	Cat# 340495
Benzonase nuclease	Sigma	Cat# E1014-25KU
Calcium chloride, 1 M	Sigma	Cat# 21115-250ML
Chloramphenicol	Sigma	Cat# C0378-25G
Corning 500ml filter system, 0.22 μM	Corning	Cat# 430758
Cystamine dihydrochloride	Sigma	Cat# C121509-25G
Cysteamine hydrochloride	Sigma	Cat# 30080-25G
Dialysis tubing cellulose membrane, 12 kDa	Sigma	D9402-100FT
DMEM medium (for growing CHO-K1 cells)	Gibco	Cat# 10938-025
DMEM medium (for growing HEK293T cells)	Sigma	Cat# D5976
DMSO	New England Biolabs	Cat# B0515A
DNA miniprep kit	Invitrogen	Cat# K210003
DNase I	Roche	Cat# 11284932001
1,2-dioleoyl-sn-glycero-3-[(N-(5-amino-1-carboxypentyl)iminodiacetic acid)succinyl] (DGS-NTA(Ni^2+^) nickel salt)	Avanti Polar Lipids	Cat# 790404P-5mg
EDTA, 1 M, pH 8	Invitrogen	Cat# 15575-038
Express PES membrane filter Unit, 0.22 μm filter	Millipore	Cat# SLGP033RS
Filtropur S, 0.45 μm	Starsted	Cat# 83.1836
Filtropur V50, 500ml, 0.2 μm	Starsted	Cat# 83.3941.001
Fluo-4, AM, cell permeant	ThermoFisher	Cat# F14201
Foetal Bovine Serum	Gibco	Cat# 10500-064 lot. 08F6480K
Freestyle CHO expression medium (for growing CHO-S cells)	Gibco	Cat# 12651-014
GeneJuice transfection reagent 5x 1mL	Merck	Cat# 70967-6
L-glutamine, 200 mM	Sigma	Cat# G7513-100ML
Glyco-diosgenin (GDN)	Generon, Anatrace	Cat# GDN101-5GM
Hepes-NaOH, 1 M	Sigma	Cat# H0887-100ML
Hepes-NaOH pH 7.5, 1 M (TCR purification)	Gibco	Cat# 15630-056
Kanamycin	Sigma	Cat# K0254-20ML
LB agar	Sigma	Cat# L7025-500TAB
LB broth	Sigma	Cat# L7275-500TAB
Magnesium chloride, 1 M	Sigma	Cat# M1028-100ML
1-palmitoyl-2-oleoyl-glycero-3-phosphocholine (POPC)	Avanti Polar Lipids	Cat# 850457P-25mg
Penicillin (5,000 U)/Streptomycin (5 mg)/Neomycin (10 mg)	Sigma	Cat# P4083-100ML
Peptide, CTag: EDQVDPRLIDGK	Genscript	Custom Peptide
Peptide, gp100: YLEPGPVTV	Genscript	Custom Peptide
Peptide, 9V: SLLMWITQV	Genscript	Custom Peptide
Phosphate buffered saline	Oxoid	Cat# BR0014G
Protease inhibitors, cOmplete EDTA-Free	Roche	Cat# 11873580001 (5056489001)
RPMI Medium 1640	Gibco	Cat# 21875-034
Silver Stain Kit	Thermo Scientific, Pierce	Cat# 24612
Sodium chloride	Sigma	S9888-1KG
Sodium pyruvate, 100 mM	Sigma	Cat# S8636-100ML
Tris-HCl pH 8.0, 1 M	Sigma	Cat# T3038-1L
Triton-X-100, 100%	Sigma	Cat# T-9284
Trypsin solution from porcine pancreas	Sigma	Cat# T4549-100ML
Urea	Sigma	Cat# U1250-1KG

**Deposited data**		

gp100/HLA-A2/TCR-CD3 complex structure	RCSB	PDB: 7PHR
gp100/HLA-A2/TCR-CD3 complex EM data	EMDB	EMD-13427

**Experimental models: Cell lines**		

Hamster: CHO-K1	Lonza	N/A
Hamster: CHO-S	Invitrogen, Gibco	Cat# R80007
Human: HEK293T	ATCC	ATCC CRL-3216
Human: Jurkat	ATCC	ATCC TIB-152
Human: THP1	ATCC	ATCC TIB-202

**Oligonucleotides**		

pEYFP reverse: ACCAGGATGGGCACCAC	IDT; Sigma	Lab ID: 554
pHR forward: TGCTTCTCGCTTCTGTTCG	IDT; Sigma	Lab ID: 1166
pHR reverse: CCACATAGCGTAAAAGGAGC	IDT: Sigma	Lab ID: 1167
pHRi forward: CAACAAGTTACCGAGAAAGAAGAACTCAC	IDT: Sigma	Lab pHRi-F-mHSP
T7 forward	Source Bioscience	In-house primers
T7 reverse	Source Bioscience	In-house primers

**Recombinant DNA**		

pHRSin_hTCRαγεζ	Addgene	ID 187351
pHRSin_hTCRαγε	Addgene	ID 187352
pHRSin_hTCRβεζ	Addgene	ID 187353
pHRSin_hTCRβε	Addgene	ID 187354
pHRSin_hCD3δ-GFP2	Addgene	ID 187355
pET28a(+)_6xHIS-hHLA-A2 (MHC Class I heavy)-C-Tag	Addgene	ID 187356
pET28a(+)_beta2M (MHC Class I light)	Addgene	ID 187357
pHRSin-IRES-EmGFP_HPC-4 Ab heavy-6xHIS	Addgene	ID 187358
pHRSin-IRES-EmGFP_HPC-4 Ab light	Addgene	ID 187359
pHRSin_GPa3b17 TCRα	Addgene	ID 187360
pHRSin_GPa3b17 TCRβ	Addgene	ID 187361
pHRi_GPa3b17 TCRα	Addgene	ID 187601
pHRi_GPa3b17 TCRβ	Addgene	ID 187602
pHRSin_1G4 hTCRα	Addgene	ID 187572
pHRSin_1G4 hTCRβ-SNAP	Addgene	ID 187573
pHRi_1G4 hTCRα	Addgene	ID 187603
pHRi_1G4 hTCRβ-SNAP	Addgene	ID 187604
pMDG	Addgene	ID 187440
P8.91	Addgene	ID 187441

**Software and algorithms**		

Calcium flux analysis code (custom)	In-house generate	https://github.com/janehumphrey/calcium
CCP4 program suite	Winn et al., 2011	RRID:SCR_007255
CHARMM-GUI	Jo et al., 2008; Lee et al., 2016	charmm-gui.org
COOT	Emsley and Cowtan, 2004	RRID:SCR_014222
cryoSPARC	Punjani et al., 2017	RRID:SCR_016501
Flowjo v10.7.1	N/A	https://www.flowjo.com
Gromacs v2020.6	Abraham et al., 2015	RRID:SCR_014565
Illustrate	Goodsell et al., 2019	github.com/ccsb-scripps/Illustrate
MDAnalysis v0.20.1	Gowers et al., 2016	github.com/MDAnalysis/mdanalysis/releases
NumPy v1.19.5	Harris et al., 2020	RRID:SCR_008633
OPM database	Lomize et al., 2012	RRID:SCR_011961
Phenix	Liebschner et al., 2019	RRID:SCR_014224
Prism v9.2.0	N/A	https://www.graphpad.com/scientific-software/prism/
PyMOL	The PyMOL Molecular Graphics System, Version 2.0 Schrödinger, LLC	RRID:SCR_000305
Rosetta	Conway et al., 2014	RRID:SCR_015701
RosettaCM	Song et al., 2013; Wang et al., 2016b	rosettacommons.org/docs/latest/application_documentation/structure_prediction/RosettaCM
Sc	Lawrence and Colman, 1993	https://www.ccp4.ac.uk/html/sc.html
Snapgene v4.1.9	N/A	https://www.snapgene.com
TOPAZ	Bepler et al., 2019	http://cb.csail.mit.edu/cb/topaz/
UCSF ChimeraX	Pettersen et al., 2021	RRID:SCR_015872
UCSF Chimera	Pettersen et al., 2004	RRID:SCR_004097
Visual Molecular Dynamics (VMD) v1.9.3	Humphrey et al., 1996	www.ks.uiuc.edu/Research/vmd/vmd-1.9.3/

**Other**		

DNA sequencing	Source Bioscience	N/A

### Resource availability

#### Lead contact

Further information and requests for resources and reagents should be directed to and will be fulfilled by the lead contact, Simon J. Davis (simon.davis@imm.ox.ac.uk).

#### Materials availability

Plasmids generated in this study have been deposited at Addgene (www.addgene.org). All other unique/stable reagents generated in this study are available from the lead contact with a completed Materials Transfer Agreement.

### Experimental model and subject details

#### Microbe strains

Rosetta 2 (DE3) pLysS bacteria were grown in suspension culture in 2.5 L Erlenmeyer flasks at 37 °C, 200 rpm in LB medium supplemented with 50 μg/mL kanamycin and 34 μg/mL chloramphenicol.

#### Cell lines

##### Hamster CHO-K1 cells

These cells were grown in adherent cell culture in T175 flasks incubated at 37 °C, 5% CO_2_ in DMEM, supplemented with 10% (v/v) FBS, 1% (v/v) Penicillin/Streptomycin/Neomycin (PSN; final concentrations 50 U/ml Penicillin, 50 μg/ml Streptomycin, 100 μg/ml Neomycin), 2 mM L-glutamine, and 1 mM sodium pyruvate.

##### Hamster CHO-S cells

CHO-S cells were maintained in suspension cell culture in 2 L Erlenmeyer flasks incubated at 37 °C, 8% CO_2_, 85% humidity, 125 rpm in Freestyle CHO Expression Medium, supplemented with 8 mM L-glutamine.

##### Human HEK-293T

HEK-293T cells were grown in adherent cell culture in T75 flasks incubated at 37 °C, 5% CO_2_ in DMEM, supplemented with 10% (v/v) FBS, 1% (v/v) PSN, and 2 mM L-glutamine.

##### Human Jurkat-derived T cells

These cells were kept in suspension cell culture in T25 flasks incubated at 37 °C, 5% CO_2_ in RPMI, supplemented with 10% (v/v) FBS, 1% (v/v) PSN, 2 mM L-glutamine, and 1 mM sodium pyruvate. The cells were maintained at a density of approximately 10^5^-10^6^ cells/ml and split the day before experiments.

##### Human THP-1 cells

THP-1 cells were maintained in suspension cell culture in T25 flasks incubated at 37 °C, 5% CO_2_ in RPMI, supplemented with 10% (v/v) FBS, 1% (v/v) PSN, 2 mM L-glutamine, 1 mM sodium pyruvate, and 10 mM Hepes, pH 7.3.

#### Cell line authentication

All the cell lines used in this study were purchased from established suppliers who authenticate the cells before distribution, except for the CHO-K1 (D28-W1) line, which was obtained from Lonza Biologics (formerly Celltech Ltd) in 1988 and for which we no longer hold records.

### Method details

#### TCR expression in CHO cells

The affinity-matured GPa3b17 TCR is encoded by the following gene segments (Liddy, 2013): the TCR-α V domain is encoded by TRAV17^∗^01 and TRAJ29^∗^01 gene segments and the C region by the TRAC^∗^01 gene segment; the TCR-β V domain is encoded by TRBV19^∗^01, TRBJ2-7^∗^01, and TRBD1^∗^01 gene segments and the C region by the TRBC1^∗^01 segment. DNA encoding full-length TCR-α and TCR-β polypeptides, and truncated forms of CD3-ε (residues 1-158), CD3-γ (1-144), CD3-ζ (1-57), and CD3-δ (1-132) fused to GFP (1-238), each separated as required by 2A ribosome-skipping sites (i.e., F2A (GSG)VKQTLNFDLLKLAGDVESNPGP, T2A (GSG)EGRGSLLTCGDVEENPGP, E2A (GSG)QCTNYALLKLAGDVESNPGP), were cloned into the pHR-SIN vector (Naldini et al., 1996) alone or in combination to create the following expression constructs: pHR-αγεζ, pHR-βεζ, and pHR-δGFP (Figure S1C). Lentivirus was made by transiently transfecting HEK-293T cells (RRID:CVCL_0063; ATCC), grown in DMEM (Sigma) supplemented with antibiotics and L-glutamine (Sigma), with the pMD.G and p8.91 packaging vectors and each of the pHR-SIN constructs, using Genejuice (Merck) according to the manufacturer’s instructions. Forty-eight to 72 hrs after transfection, the supernatants were harvested and 0.2 μm filtered (Millipore) to remove cell debris. 1 x 10^6^ Freestyle Chinese hamster ovary cells (CHO-S; RRID:CVCL_7183; ThermoFisher) in 1 mL of Freestyle CHO Expression Medium (Gibco) supplemented with L-glutamine, were then transduced overnight with 2 mL of pHR-αγεζ and pHR-βεζ lentiviruses and 1 mL of the pHR-δGFP lentivirus. The cells were recovered with 5 mL of Freestyle CHO Expression Medium supplemented with L-glutamine the next day and grown as attached cells. Four days later, 1 x 10^6^ of these cells were re-transduced with the expression constructs before being converted back to suspension cells via culturing in an orbital shaker at 125 rpm and 37 °C. Expression was confirmed after seven days using flow cytometry, by incubating the TCR-expressing cells with gp100/HLA-A2 tagged with Alexa Fluor 647 (Molecular Probes), and/or PE-labelled UCHT1 antibody (Biolegend) for 30 min at 4 °C in the dark. All flow cytometric analysis was conducted on an Attune NxT flow cytometer (ThermoFisher), and the data analyzed with FlowJo software.

#### Preparation of HPC-4 epitope-tagged soluble gp100/HLA-A2, and anti-HPC-4 resin

DNA encoding residues 25-304 of the heavy chain of HLA-A2, flanked by six histidine residues at the N-terminus and by an HPC-4 epitope tag (‘C-Tag’: EDQVDPRLIDGK) at the C terminus, and residues 22-119 of the light chain (β_2_-microglobulin) were each cloned into the pET28a(+) vector (Novagen) to yield the pET28a(+)-HLA-A2(Heavy) and pET28a(+)-HLA-A2(Light) expression vectors. Expression of each of the polypeptides and refolding of HPC-4 tagged HLA-A2 in the presence of gp100 peptide (YLEPGPVTV), were undertaken as described (Garboczi et al., 1992). Briefly, inclusion bodies released by cell disruption at 28,000 psi, 10 °C (Constant Systems) were centrifuged at 15,000*g* for 10 min at 4 °C. The washed, pelleted inclusion bodies were solubilized in 50 mM Tris/HCl pH 8.0, 10 mM EDTA, 8 M urea overnight at room temperature, before being clarified by centrifugation at 15,000*g* for 10 min at 4 °C and 0.2 μm filtration (Millipore) and concentrated to 15 mg/mL. Seventy-five milligrams of the MHC heavy chain inclusion bodies and 30 mg of light chain inclusion bodies in 50 mM Tris/HCl pH 8.0, 100 mM NaCl, 8 M urea, were denatured with 10 mM DTT at 37 °C for 30 min. The two polypeptides, along with 15 mg of gp100 peptide in DMSO, were then added to 100 mM Tris/HCl pH 8.0, 2 mM EDTA, and 1.2 M L-arginine in 12 kDa MWCO cellulose dialysis tubing (Sigma) and dialyzed against de-ionized water (1 change after 72 h) and then 10 mM Tris/HCl pH 8.0 (after 80 h). After 96 h, the soluble gp100/HLA-A2 was concentrated using a Vivaflow 200, 10 kDa MWCO PES concentrator and Amicon Ultra-15 10 kDa MWCO Ultracel-10 membrane (Millipore) and purified by SEC on a Superdex 200 HiLoad 16/600 GL column (GE Healthcare) pre-equilibrated with PBS pH 8.0 supplemented with 0.05% (w/v) sodium azide. The gp100/HLA-A2-containing fractions were pooled and concentrated to 1 mg/mL.

DNA encoding anti-HPC-4 antibody heavy and light chains (Rezaie and Esmon, 1995), codon-optimized for expression in CHO cells was cloned into separate pHR-IRESEm plasmids; a 6x histidine tag at the C terminus of the heavy chain was also encoded to facilitate antibody purification. Lentiviruses were generated by separate transient transfections of HEK-293T cells of each construct using Genejuice. Forty-eight to 72 h after transfection, two milliliters of the 0.2 μM filtered supernatant containing lentiviruses was used to transduce 1 x 10^6^ CHO-K1 cells overnight. After four days recovery in 5 mL DMEM supplemented with 10% FBS (Gibco), antibiotics and 2 mM L-glutamine, the supernatant was removed, and the cells used to seed large-scale culture for supernatant harvesting every 4-5 days. Anti-HPC-4 antibody was affinity purified by nickel-chelation chromatography (Qiagen). Absorbance at 280 nm and 12% acrylamide Coomassie Blue-stained analytical gels were used to confirm antibody quality. Sixteen liters of supernatant yielded 1g of antibody. The antibody was buffer exchanged into 20 mM Hepes pH 7.4, 150 mM NaCl, 0.05% (w/v) sodium azide using SnakeSkin dialysis tubing (Thermo Scientific) before being coupled to CNBr-activated Sepharose according to the manufacturer’s instructions (GE Healthcare).

#### TCR purification

CHO-S cells expressing the TCR were grown in Freestyle CHO Expression Medium supplemented with L-glutamine in 1 L cultures in 2.5 L Erlenmeyer flasks (Corning) and harvested when they reached a density of 2.8 x 10^6^ cells/mL. The cells were recovered by centrifugation at 500*g* for 10 min at 4 °C. The cells were then washed with cold PBS and re-pelleted before being resuspended and an aliquot stained with gp100/HLA-A2 for 1 h at 4 °C to allow sustained expression to be confirmed by flow cytometry. Cells were washed with cold PBS and pelleted for storage at -80 °C.

Cells harvested from a total of 10-50 L of culture were re-suspended in 30 mM Tris/HCL pH 8.0, 750 mM NaCl supplemented with Roche cOmplete™ Protease Inhibitor Cocktail, 2.5 U/μL Benzonase Nuclease (Sigma) and 10 μg/mL DNase I (Roche), and disrupted at 5,000 psi using a benchtop 1.1-KW cell disruptor (Constant Systems), cooled to 10 °C with a Frigomix R (Sartorius-Stedim). The cell lysate was centrifuged at 600*g* for 10 min at 4 °C. The supernatant was transferred to 70 mL Beckman Coulter centrifuge tubes (cat. 355622) and centrifuged using a Type 45 TI rotor at 15,000*g* for 5 min at 4 °C. The supernatant was collected and re-centrifuged at 100,000*g* for 60 min at 4 °C to pellet the membranes. The membrane fraction was solubilized into 20 mM HEPES pH 7.5 (Gibco), 500 mM NaCl, 15% (v/v) glycerol (MP Biomedicals) supplemented with Roche cOmplete™ Protease Inhibitor Cocktail, 1% (w/v) GDN (Anatrace), 2.5 U/μL Benzonase Nuclease, 1 mM CaCl_2_, by rotation at 5 rpm overnight at 4 °C. The soluble fraction was isolated by centrifugation at 142,000*g* for 4 h at 4 °C, 5 μm and 0.45 μm filtered (Sartorius), and passed over 2 mL of anti-HPC-4 antibody-coupled Sepharose beads in a 10 mL column (VWR) pre-equilibrated with solubilization buffer without detergent. The resin was washed with 20 mM Hepes pH 7.5, 500 mM NaCl supplemented with 1 mM CaCl_2_, 2 mM ATP (Enzo Life Sciences), 0.15% (w/v) GDN, and 20 mM Hepes pH 7.5, 500 mM NaCl, 1 mM CaCl_2_, 2 mM ATP, and 0.05% (w/v) GDN. The TCR complex was eluted using 200 μg/mL C-Tag peptide (Ne Biotech) in elution buffer comprised of 20 mM Hepes pH 7.5, supplemented with 500 mM NaCl, 1 mM EDTA, and 0.05% (w/v) GDN. Finally, the eluted complex was purified by SEC on a Superdex 200 HiLoad 16/600 GL column (GE Healthcare) in the elution buffer. Analytical, high-resolution SEC was performed using an ACQUITY UPLC Protein BEH200 SEC column (Waters).

#### Signaling activity of the GPa3b17 TCR

Tests of GPa3b17 TCR-induced calcium signaling were undertaken using SLBs. Glass coverslips (25 mm, thickness no. 1.5; VWR) were prepared by incubation in a 3:1 mixture of pure sulfuric acid/hydrogen peroxide (30% solution in water, Merck) at room temperature overnight, then rinsed thoroughly in MQ water, dried rapidly and plasma cleaned for 1 min. SLBs were formed by vesicle fusion in CultureWell 50-well silicon covers (Grace Bio-Labs) which were cut to size and placed on the cleaned coverslips. Small unilamellar vesicle (SUV) lipid preparations were produced by mixing 98% POPC and 2% DGS-NTA-Ni^2+^ (Avanti Polar Lipids) molar solutions in chloroform in a 1.5 mL amber glass vial (Merck) and dried under a stream of nitrogen. The lipids were then resuspended in 0.22 μm filtered PBS to a final concentration of 1 mg/mL, vortexed for 30 s and tip sonicated on ice for 30 min. Five microliters of the SUV mixture was added to each well with 5 μL of filtered PBS and incubated at room temperature for 30 min. Each well was then washed five times with 5 μL of filtered PBS before adding 5 μL of protein mixture and incubating for 1 h at room temperature. Refolded and purified gp100/HLA-A2 was produced with 2 x 6 histidine C-terminal tags as described above for binding to the bilayers. Protein mixtures were prepared with the gp100/HLA-A2 at 0.2 μg/mL final concentration and a ‘null’, irrelevant pMHC (HLA-A2 presenting the 9V peptide of NY-ESO; Chen et al., 2005) at a final concentration of 10 μg/mL to saturate all available nickel sites on the SLB. Immediately before use, each bilayer was washed 10 times with 5 μL filtered PBS.

To prepare the cells for calcium signaling experiments, 1 x 10^6^ GPa3b17 TCR-expressing Jurkat T cells were placed in a 1.5 mL Eppendorf tube, pelleted, and resuspended in 100 μL RPMI, 100 μL HBS, and 1 μL Fluo-4 AM calcium dye (25 μg/mL final concentration; ThermoFisher). The cells were then incubated at 37 °C and 5% CO_2_ for 5 min before being pelleted and washed twice in 500 μL PBS. The cells were finally resuspended in 200 μL pre-warmed PBS before imaging. Approximately 5 μL of the cell suspension was added to each SLB-containing well for imaging on a Zeiss LSM 880 Inverted Microscope with Airyscan (Carl Zeiss), using a 10x objective to capture several hundred to a thousand cells in the field of view. Fluo-4 was excited using an Argon 488 nm laser at 10% power and images were taken every second for a total of 10 min without averaging. The fraction of cells triggering under each condition was analyzed by a bespoke MATLAB® script (written by Jane Humphrey and Aleks Ponjavic; Klenerman Laboratory, Department of Chemistry, Cambridge). Comparisons were made with Jurkat T cells expressing the 1G4 TCR that interacts with SLBs loaded with soluble HLA-A2 presenting the 9V (SLLMWITQV) NY-ESO peptide (Chen et al., 2005).

Tests of intermediate signaling induced by the affinity matured GPa3b17 TCR were undertaken by measuring CD69 upregulation. GPa3b17 TCR-expressing Jurkat T cells were incubated overnight with wild-type THP-1 APCs (ATCC) and THP-1 cells whose expression of β_2_-microglobulin was prevented using CRISPR/Cas9 targeting, in the presence and absence of 100 μM gp100 peptide. CD69 expression was then analyzed by flow cytometry.

#### Cryo-EM sample preparation and data collection

Purified TCR was concentrated to 2 mg/mL, mixed with UCHT1 Fab (1:5 molar ratio), and incubated for 30 min on ice. Three microliters of protein solution was deposited on a graphene-coated UltraAuFoil R1.2/1.3 grid (Quantifoil). Excess protein was blotted away for six seconds with a force of 25 at 4 °C and 100% relative humidity before plunge-freezing in liquid ethane using a Vitrobot Mark IV (Thermo Scientific). EZ transfer graphene was obtained commercially (Graphenea) and deposited onto the grid by floating in a water bath, and the sacrificial polymer layer removed according to the manufacturer’s instructions. 50 nM 1-pyrenecarboxylic acid in isopropanol was used to increase hydrophilicity (D'Imprima et al., 2019) before rinsing with pure isopropanol and air drying. Despite a reduction in image contrast, use of graphene proved crucial for stabilizing the TCR, as vitrification on unsupported grids did not yield sufficiently intact particles. However, graphene alone was insufficient to adequately stabilize the TCR for high resolution imaging, which was only achieved with the addition of UCHT1 Fab. It should be noted that virtually no density for UCHT1 Fab was observed in the final cryo-EM reconstruction, even though biochemical analysis confirmed UCHT1 binding in a 2:1 stoichiometry (Figures S1E–S1G), and weak density associated with the Fab could be discerned in reference-free 2D classifications of the gp100/HLA-A2/TCR particles (Figure S2G). Thus, dominant contributions of UCHT1 to particle alignment seem unlikely. Rather, we speculate that binding of UCHT1 Fab to the peripherally located CD3-ϵ subunits shielded the TCR from unfavorable interactions with the air-water or graphene-water interfaces enabling high-resolution structure determination. Vitrified grids were imaged in a Titan Krios (FEI) equipped with a K2 Summit direct electron detector operating in counting mode. Zero-loss imaging was performed with a 20 eV slit width using a GIF Quantum SE post-column energy filter (Gatan). Micrographs were collected in SerialEM (Mastronarde, 2005) as 70-frame movie stacks at a nominal magnification of 130,000×, corresponding to a calibrated pixel size of 1.05 Å/pixel at the specimen level. Cryo-EM data collection parameters are summarized in Table S1.

#### Cryo-EM data processing

Movie stacks were imported into cryoSPARC (Punjani et al., 2017) and subjected to Patch Motion correction followed by Patch CTF-estimation. A subset of 100 randomly selected micrographs was used to train a picking model in TOPAZ (Bepler et al., 2019), which was subsequently applied to the entire dataset. TOPAZ-picked particles were extracted with a box size of 384 pixels and subjected to multi-class ab initio reconstruction (class similarity 0). The ab initio map corresponding to the lone ‘good’ class, with clear protein features, as well as two ‘junk’ maps, lacking discernable protein features, were selected as references for downstream hetero refinement of the entire particle stack. Hetero refinement was then iterated until 98% of the remaining particles classified into the good class. The final image stack of 154,408 particles was subsequently subjected to non-uniform refinement and CTF refinement in cryoSPARC resulting in a cryo-EM map at 3.08 Å resolution. The cryo-EM processing workflow is illustrated in Figure S2B .

#### Model building and refinement

An initial model was generated using RosettaCM (Song et al., 2013; Wang et al., 2016b) with PDB entries 1SY6, 1XIW, 5EU6, and 6JXR as starting templates. All non-protein components and antibody fragments were removed from the starting models prior to density-guided rebuilding in RosettaCM against the final cryo-EM map in real space. Rosetta scripts were adapted from the DiMaio Lab repository (University of Washington, https://dimaiolab.ipd.uw.edu/software/). The highest ranked Rosetta models by geometry and density scores were visually inspected and manually adjusted in COOT (Emsley and Cowtan, 2004). Iterative rebuilding in COOT was followed by density-guided relaxation in Rosetta (Conway et al., 2014). Finally, real-space refinement was performed using Phenix (Liebschner et al., 2019). The refinement strategy included global minimization, local grid search, and ADP refinement, with default and secondary-structure restraints enabled. Refinement and validation statistics are summarized in Table S1.

#### Molecular dynamics simulations

We performed molecular dynamics simulations of four simulation systems (A–D). System A (GPa3b17 partial) comprised gp100/HLA-A2 in complex with a truncated model of the high affinity GPa3b17 TCR, comprising TCR-α residues 9-201 and TCR-β residues 2-243. System B [wild-type (WT) partial] comprised gp100/HLA-A2 in complex with a truncated model of the parental WT TCR, comprising TCR-α residues 9-201 and TCR-β residues 2-243, with the following residues mutated to represent the parental TCR: αS95D, αM98L, αQ99V, βW51Q, βA52I, βQ53V, βG54N, βW96I, βA98G. System C (GPa3b17 full) comprised the fully assembled high-affinity gp100/HLA-A2/GPa3b17 TCR model as determined in this work, embedded in an asymmetric lipid bilayer representing the plasma membrane, following Kalli et al. (2017). The outward-facing leaflet had membrane composition POPC (33.3 mol%), PSM (33.3 mol%), and cholesterol (33.3 mol%); the cytosolic leaflet had membrane composition POPC (35 mol%), POPE (25 mol%), POPS (20 mol%), and cholesterol (20 mol%). System D (WT full) comprised the fully assembled gp100/HLA-A2/GPa3b17 TCR model as determined in this work, with mutations in TCR-α and TCR-β as in system (B), and a lipid membrane composition as in (C).

All simulations were performed with GROMACS 2020.6 (Abraham et al., 2015) using the CHARMM36m forcefield (Huang et al., 2017) for all solutes in combination with the TIP3P water model. We set up the simulation systems with CHARMM-GUI (Jo et al., 2008; Lee et al., 2016). All systems were solvated in a water box of starting dimensions 15.7 x 15.7 x 15.7 nm^3^ for systems A and B, and 13.3 x 13.3 x 22.5 nm^3^ for systems C and D. The lipid bilayer in C and D was orientated in the xy plane. We added Na^+^ and Cl^-^ ions at 150 mM concentration. We energy-minimized all systems by steepest descent until convergence (tolerance: 1000 kJ/[mol nm]). All systems were first equilibrated in the *NVT* ensemble for 125 ps. For all simulations, the temperature was maintained at 300 K using the v-rescale thermostat (Bussi et al., 2007) with τ_t_ = 1 ps. Following the *NVT* equilibration, we equilibrated the systems in the *NPT* ensemble for 1.25 ns. The pressure was maintained at 1 bar using isotropic pressure coupling (compressibility K = 4.5x10^-5^ bar^-1^) with the Berendsen barostat (Berendsen et al., 1984; τ_p_=1 ps) for systems A and B, and semi-isotropic pressure coupling for systems C and D. We performed production simulations in the *NPT* ensemble for 1 μs, with pressure maintained at 1 bar using the Parrinello-Rahman barostat (Parrinello, 1981) with τ_p_ = 5 ps. The first 100 ns of the production simulations were discarded as further equilibration. For equilibration simulations, we applied position restraints to the protein heavy atoms and, for systems C and D, lipid heavy atoms. For production simulations, no external restraints were applied. We used the python packages *MDAnalysis* (Gowers, 2016) and *NumPy* (Harris et al., 2020) for the analysis of the protein systems.

#### Analysis of the pMHC/TCR interface

The shape complementarity was calculated with the CCP4 program sc (Winn et al., 2011). UCSF ChimeraX (Pettersen et al., 2021) was employed to determine the buried surface area, using the default probe radius of 1.4 Å.

#### Figure preparation

Structure figures were prepared using UCSF Chimera (Pettersen et al., 2004) and PyMOL (The PyMOL Molecular Graphics System, Version 2.0 Schrödinger, LLC), except for Figure 7, which was prepared with the program Illustrate (Goodsell et al., 2019), using membrane coordinates from the OPM (Lomize et al., 2012; PDB ID: 6JXR). To depict the CD2-CD58 complex, structures of the CD2 ectodomain (PDB ID: 1HNF) and of a CD58 (domain 1)/CD2 (domain 2) chimera (PDB ID: 1CCZ) were superimposed onto the structure of the CD2/CD58 complex comprising the N-terminal domains only (PDB ID: 1QA9). To illustrate CD8αβ binding, the MHC α3 domain of the CD8αβ-H-2D^d^ complex structure (PDB ID: 3DMM) was superimposed onto the same domain of the gp100/HLA-A2/GPa3b17 TCR complex. To analyze the position of the CD8 binding site in the canonical and reversed pMHC I/TCR docking polarities, TCR-αβ of the B17.C1-pMHC I (PDB ID: 7JWJ), B17.R1-pMHC I (PDB ID: 5SWZ), and B17.R2-pMHC I (PDB ID: 7JWI) structures were superimposed onto TCR-αβ of our gp100/HLA-A2/GPa3b17 TCR complex.

### Quantification and statistical analysis

The data shown in Figures S1A and S1B are representative of 3-4 replicate experiments. Reported resolutions of the cryo-EM map are based upon the 0.143 Fourier Shell Correlation criterion. Model validation statistics (Table S1) were computed as implemented in MolProbity (Davis et al., 2007) of the Phenix software package (Liebschner et al., 2019).

## Data Availability

• The cryo-EM density map has been deposited at the Electron Microscopy Data Bank (https://www.ebi.ac.uk/emdb/) and its associated model coordinates have been deposited at the Protein Data Bank (https://www.ebi.ac.uk/pdbe/) and are publicly available as of the date of publication. Accession numbers are listed in the key resources table. • This study did not generate new code. • Any additional information required to re-analyze the data reported in this paper is available from the lead contact upon request.

## Supplemental information

Table S1. Cryo-EM data collection, refinement, and validation statistics, related to Figure 1
