# Supplementary material for: Structure of a fully assembled tumor-specific T cell receptor ligated by pMHC
Source: Cell. 2022 Aug 18;185(17):3201–3213.e19. doi: 10.1016/j.cell.2022.07.010 (PMC9630439; doi:10.1016/j.cell.2022.07.010)
Supplement: Table S1. Cryo-EM data collection, refinement, and validation statistics, related to Figure 1 [file mmc1.pdf]

Cell, Volume *185*

Supplemental information

Structure of a fully assembled tumor-specific T cell receptor ligated by pMHC

Lukas Sušac, Mai T. Vuong, Christoph Thomas, Sören von Bülow, Caitlin O'Brien-Ball, Ana Mafalda Santos, Ricardo A. Fernandes, Gerhard Hummer, Robert Tampé, and Simon J. Davis

Table S1 | Cryo-EM data collection, refinement, and validation statistics

	**gp100/HLA-A2/TCR-CD3 complex** (EMDB-13427; PDB 7PHR)
**Data collection and processing**	
Microscope	TITAN-Krios
Camera	Gatan K2 Summit
Magnification	130,000
Voltage (kV)	300
Electron exposure (e–/Å^2)	62
Defocus range (μm)	-1.2 to -2.2
Pixel size (Å)	1.05
Symmetry imposed	C1
Initial particle images (no.)	450,996
Final particle images (no.)	154,408
Map resolution (Å)	3.08
FSC threshold	0.143
Map resolution range (Å)	2.61 to 5.05
**Refinement**	
Map sharpening *B* factor (Å^2)	-78.6
Model composition	
Non-hydrogen atoms	11,511
Protein residues	1,443
Ligands	6 (NAG)
B factors (Å^2)	
Protein	41.97
Ligands	43.07
R.m.s. deviations	
Bond lengths (Å)	0.0055
Bond angles (°)	0.77
Validation	
MolProbity score	1.41
Clashscore	5.35
Poor rotamers (%)	0.08
Ramachandran plot	
Favored (%)	97.40
Allowed (%)	2.60
Disallowed (%)	0.00
